# ATG8ylation of vacuolar membrane protects plants against cell wall damage

**DOI:** 10.1038/s41477-025-01907-z

**Published:** 2025-02-07

**Authors:** Jose Julian, Peng Gao, Alessia Del Chiaro, Juan Carlos De La Concepcion, Laia Armengot, Marc Somssich, Heloise Duverge, Marion Clavel, Nenad Grujic, Roksolana Kobylinska, Ingo Polivka, Maarten Besten, Tonni Grube Andersen, Christian Dank, Barbara Korbei, Andreas Bachmair, Nuria S. Coll, Elena A. Minina, Joris Sprakel, Yasin Dagdas

**Affiliations:** 1 Gregor Mendel Institute, Austrian Academy of Sciences, Vienna BioCenter, Vienna, Austria; 2 Department of Applied Genetics and Cell Biology, Institute of Molecular Plant Biology, BOKU University, Vienna, Austria; 3 Max Perutz Labs, Department of Biochemistry and Cell Biology, University of Vienna, Vienna, Austria; 4 Vienna BioCenter PhD Program, Doctoral School of the University of Vienna and Medical University of Vienna, Vienna, Austria; 5 Centre for Research in Agricultural Genomics, CSIC-IRTA-UAB-UB, Bellaterra, Spain; 6 Max Planck Institute for Plant Breeding Research, Cologne, Germany; 7 Institute of Organic Chemistry, University of Vienna, Vienna, Austria; 8 Laboratory of Biochemistry, Wageningen University & Research, Wageningen, the Netherlands; 9 Consejo Superior de Investigaciones Científicas, Barcelona, Spain; 10 Department of Molecular Sciences, Uppsala BioCenter, Swedish University of Agricultural Sciences and Linnean Center for Plant Biology, Uppsala, Sweden; 11 Present Address: Centre for Organismal Studies, Heidelberg University, Heidelberg, Germany; 12 Present Address: Max Planck Institute of Molecular Plant Physiology, Potsdam, Germany

**Keywords:** Plant cell biology, Stress signalling

## Abstract

Vacuoles are essential for cellular metabolism and growth and the maintenance of internal turgor pressure. They sequester lytic enzymes, ions and secondary metabolites that, if leaked into the cytosol, could lead to cell death. Despite their pivotal roles, quality control pathways that safeguard vacuolar integrity have remained elusive in plants. Here we describe a conserved vacuolar quality control pathway that is activated upon cell wall damage in a turgor-pressure-dependent manner. Cell wall perturbations induce a distinct modification—ATG8ylation—on the vacuolar membrane (tonoplast) that is regulated by the V-ATPase and ATG8 conjugation machinery. Genetic disruption of tonoplast ATG8ylation impairs vacuolar integrity, leading to cell death. Together, our findings reveal a homeostatic pathway that preserves vacuolar integrity upon cell wall damage.

## Abstract (web summary)

ATG8ylation of the tonoplast, triggered by cell wall damage, acts as a vital vacuolar quality control mechanism that safeguards vacuolar integrity and ensures cell survival under stress.

## Main

Plant cells are pressurized compartments, encased within a rigid wall that must simultaneously resist turgor pressure and accommodate growth through flexibility^1,2^. The cell wall, a dynamic matrix of structural polysaccharides including cellulose, hemicelluloses and pectin, along with a suite of structural and modulatory proteins, is critical for defining cell morphology and maintaining cellular integrity^3,4^. Moreover, it serves as the primary defence barrier against pathogens and abiotic stresses^4–6^. Consistent with its role in cellular viability and function, plants have evolved elaborate cell wall integrity (CWI) pathways that closely surveil disruptions in wall composition and mechanics to initiate homeostatic mechanisms that restore CWI^2,6^. So far, CWI studies have mainly focused on cell wall homeostasis. The impact of cell wall damage on cellular organelles and the contribution of these organelles to maintaining CWI remain largely unknown^4^.

Vacuoles account for up to 80% of the cellular volume^7,8^. The expansion of the vacuole within the inflexible cell wall generates turgor pressure that is characteristic of plant cells and is required for growth^9–11^. The receptor-like kinase FERONIA, a key sensor of CWI, has been implicated in the modulation of vacuolar morphology during cell expansion^9^, suggesting a potential link between CWI sensing and vacuolar integrity. Furthermore, CWI signalling is sensitive to osmotic fluctuations; for instance, osmolytes such as sorbitol can dampen CWI responses^12^. A pressing question that remains unexplored is how plants preserve vacuolar integrity when faced with sudden changes in cell wall structure. Without the protection of the rigid cell wall, the turgor pressure imbalance could lead to the rupture of the tonoplast and cell death^13^. Identifying quality control pathways that safeguard vacuolar integrity is therefore essential to improving plant resilience and adaptability.

A hallmark of macroautophagy (hereafter autophagy) is the conjugation of the ubiquitin-like protein ATG8 to the lipid phosphatidylethanolamine and its subsequent insertion into the double-membrane phagophore via the combined action of conserved autophagy-related (ATG) proteins^14^. ATG8 conjugation to the phagophore membrane involves a ubiquitination-like cascade. First, ATG8 gets activated by the ATG4 protease, exposing the carboxy-terminal glycine residue^15^. Processed ATG8 is activated by the E1-like enzyme ATG7, transferred to the E2-like enzyme ATG3 and conjugated to the phagophore membrane by the E3-like enzyme ATG5–ATG12–ATG16 (ref. ^15^). Here ATG16 is particularly important, because it has an ability to bind membranes and thereby defines the site of ATG8 lipidation^15^. While investigating how cell wall damage influences autophagy, we unexpectedly discovered that cell wall damage triggers the conjugation of ATG8 to the single-membrane tonoplast in both *Arabidopsis thaliana* and *Marchantia polymorpha*. Genetic characterization of this conjugation of ATG8 to single membranes (CASM) process reveals the V-ATPase–ATG16 axis as a key module regulating tonoplast ATG8ylation. Ultrastructural analysis of CASM-deficient plants shows that tonoplast ATG8ylation is essential for vacuolar integrity and cell viability. Altogether, our findings reveal that ATG8ylation is a vacuolar quality control (VQC) mechanism that protects vacuolar integrity upon cell wall damage.

## Results

### Cell wall damage triggers ATG8ylation of the tonoplast

**Fig. 1:**
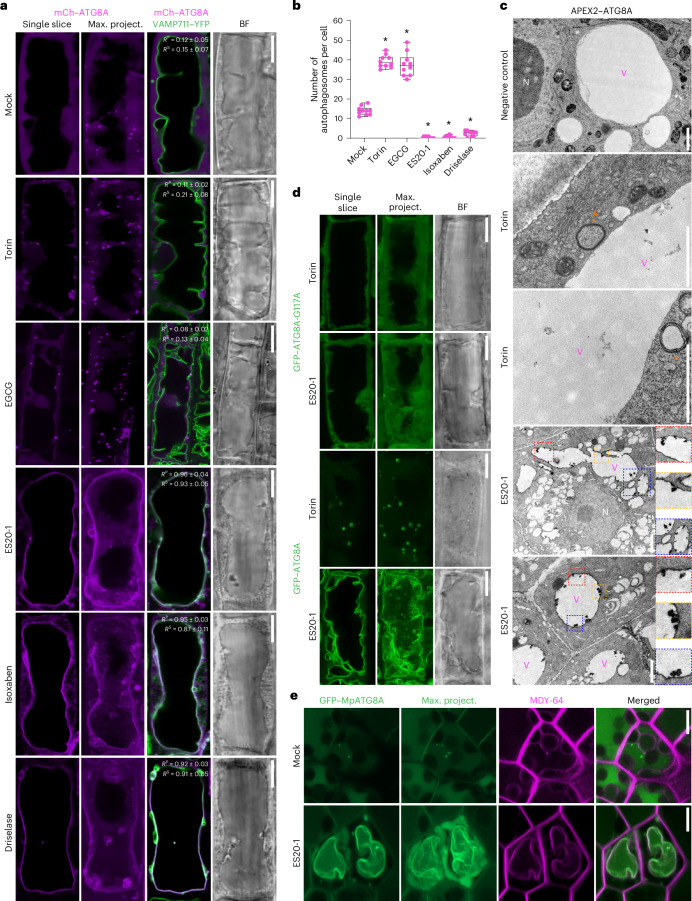
Cell wall damage induces ATG8ylation of the tonoplast. **a**, Confocal micrographs of root cells in the early elongation zone of *A. thaliana*, showing mCherry–ATG8A (mCh–ATG8A, magenta) to illustrate the relocalization of ATG8 to the tonoplast upon cell wall damage. A single optical slice and a maximum intensity projection (max. project.) of a whole cell (20 µm depth), alongside a merged image with VAMP711–YFP (tonoplast marker) and a corresponding bright field (BF) image, are shown. Pearson and Spearman colocalization values indicate the association between ATG8A and the tonoplast. The treatment conditions include mock, Torin (1.5 h, 9 µM), EGCG (30 mins, 50 µM), ES20-1 (8 h, 100 µM), isoxaben (3 days, 3 nM) and Driselase (1 h, 1%). Scale bars, 10 µm. **b**, Quantification of autophagosomes under the treatment conditions depicted in **a**. One-sided Wilcoxon tests compared the treatments (*n* = 10) to mock; significant differences (*P* < 0.01) are indicated with asterisks. In each box plot, the central line indicates the median, and the upper and lower bounds represent quartile 3 (75th percentile) and quartile 1 (25th percentile), respectively. The whiskers denote the minima and maxima of the data points. **c**, Electron microscopy images displaying APEX2–ATG8A localization after Torin (1.5 h, 9 µM) or ES20-1 treatments (8 h, 100 µM), with (mock) or without DAB staining (negative control). The Torin-treated samples show typical autophagosome structures, whereas ES20-1 treatments lead to the labelling of the tonoplast. The insets show densely labelled tonoplast invaginations. N, nucleus; V, vacuole; A, autophagosome (orange arrowheads). Representative images from three seedlings were analysed under each treatment. Scale bars, 1 µm. **d**, Confocal micrographs of *A. thaliana* root cells expressing the GFP–ATG8A-G117A mutant, which is incapable of conjugating to membranes. Images are shown after treatment with Torin (1.5 h, 9 µM) or ES20-1 (8 h, 100 µM). Representative images from ten seedlings were analysed under each treatment. Scale bars, 10 µm. **e**, Confocal micrographs of *M. polymorpha*, comparing GFP–ATG8A localization under mock or ES20-1 (8 h, 100 µM) treatment conditions. MDY-64 (1 h, 1 µM) staining is used to mark tonoplast localization. Representative images from ten gemmae were analysed under each treatment. Scale bars, 10 µm.

**Extended Data Fig. 1:**
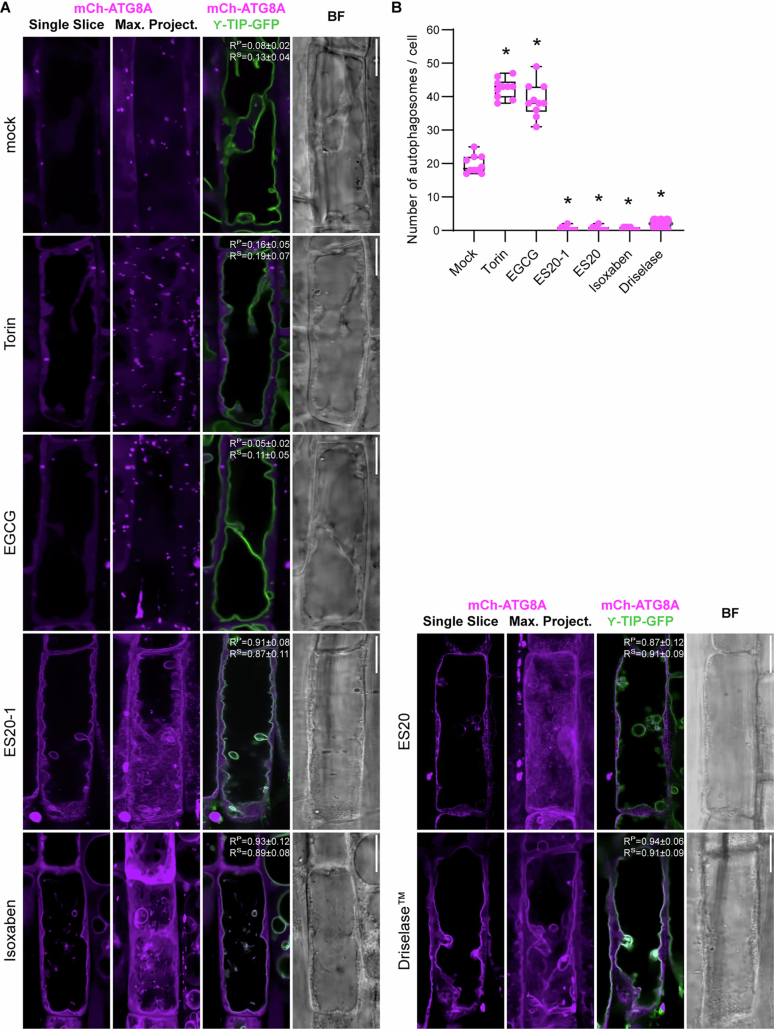
Cell wall damage triggers ATG8ylation of the tonoplast. **(a)** Confocal micrographs of the early elongation zone root cells of *Arabidopsis thaliana*, depicting the co-localization of mCherry-ATG8A (magenta) with the tonoplast marker *γ*-tip-GFP. The set includes a single optical slice and a maximum intensity projection of an entire cell (20 µm depth), along with a merged image incorporating *γ*-tip-GFP and a corresponding bright field image. Scale bar, 10 µm. Pearson and Spearman co-localization scores are provided to quantify the association between ATG8A and the tonoplast, under treatment conditions including mock, Torin (1.5 hours, 9 µM), EGCG (30 minutes, 50 µM), ES20 (8 hours, 100 µM), ES20-1 (8 hours, 100 µM), Isoxaben (3 days, 3 nM), and Driselase (1 hour, 1%). **(b)** Quantitative analysis of autophagosome numbers under the treatment conditions shown in Supplementary Fig. 1a. One-sided Wilcoxon test compared treatments (n = 10) to mock; significant differences (p < 0.01) are indicated as asterisks. The central line indicates the median, and the upper and lower bounds represent quartile 3 (75th percentile) and quartile 1 (25th percentile), respectively. The whiskers denote the minima and maxima of the data points.

To investigate whether cell wall damage triggers autophagy, we performed confocal microscopy analysis on ATG8 reporter lines upon cell wall damage. First, we tested the colocalization of mCherry–ATG8A with the tonoplast markers VAMP711–YFP and γ-TIP–GFP under control conditions. Few autophagic puncta that we detected did not colocalize with the tonoplast under basal conditions (Pearson’s colocalization value (*R*^P^), 0.12 ± 0.05; Spearman’s colocalization value (*R*^S^), 0.15 ± 0.07) (Fig. 1a and Extended Data Fig. 1a). We then used the TOR kinase inhibitor Torin1 (hereafter Torin) to induce bulk autophagy^16^. Torin increased the number of autophagic puncta, but these puncta did not colocalize with the tonoplast (*R*^P^ = 0.11 ± 0.02, *R*^S^ = 0.21 ± 0.08) (Fig. 1a,b and Extended Data Fig. 1).

**Extended Data Fig. 2:**
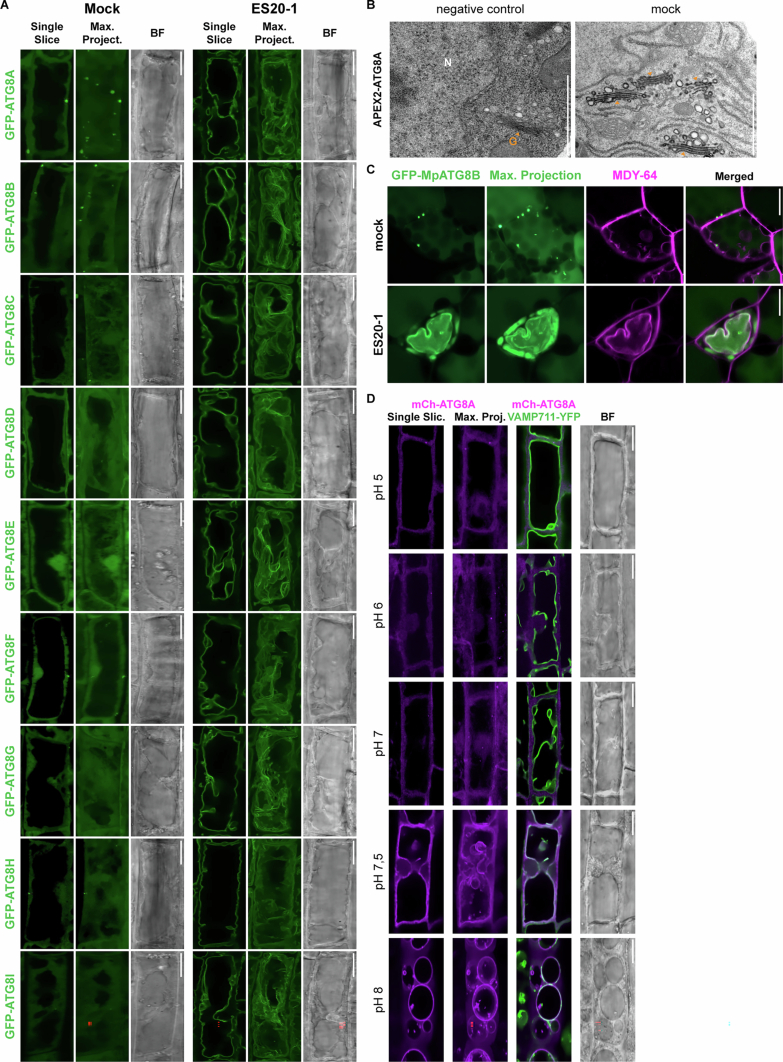
All Arabidopsis and Marchantia ATG8 isoforms are recruited to the tonoplast upon cell wall damage. **(a)** Confocal micrographs displaying the localization of all nine GFP-tagged ATG8 isoforms (GFP-ATG8A to GFP-ATG8I) in *Arabidopsis thaliana* root cells. For each isoform, images under mock conditions include a single optical slice, a maximum intensity projection of an entire cell (20 µm depth), and a corresponding bright field image. The same set of images is presented for cells treated with ES20-1 (8 hours, 100 µM). Representative image from 10 seedlings analyzed under each treatment. Scale bar, 10 µm. **(b)** Electron microscopy (EM) images displaying APEX2-ATG8A localization post DAB staining to highlight unspecific labeling of the Golgi stacks in APEX2-ATG8A lines. N: Nucleus, G and orange arrowheads: Golgi apparatus. Representative image from 3 seedlings analyzed under each treatment. Scale bar, 1 µm. **(c)** Confocal micrographs of *Marchantia polymorpha*, comparing GFP-ATG8B localization under mock or ES20-1 (8 hours, 100 µM) treatment conditions. MDY-64 (1 hour, 1 µM) staining is used to mark tonoplast localization. Representative image from 10 gemmae analyzed under each treatment. Scale bar, 10 µm. **(d)** Alkaline pH treatment induces ATG8ylation of the tonoplast. Confocal micrographs of root cells in the early elongation zone of *Arabidopsis thaliana*, highlighting the localization of mCherry-ATG8A (magenta) to illustrate re-localization of ATG8 to the tonoplast upon alkaline stress. The panel includes a single optical slice and a maximum intensity projection of a whole cell (20 µm depth), alongside a merged image with VAMP711-YFP (tonoplast marker) and a corresponding bright field image. Treatment conditions include pH 5, 6, 7, 7,5 and 8 for 3 hours. Representative image from 10 seedlings analyzed under each treatment. Scale bar, 10 µm.

To test the effect of cell wall damage on autophagy, we first performed pectin methylesterase inhibitor epigallocatechin gallate (EGCG) treatment. EGCG increases cell wall stiffness by changing pectin methylation and polymerization^9^. EGCG treatment increased the number of autophagic puncta, and, similar to Torin treatment, they did not colocalize with the tonoplast (*R*^P^ = 0.08 ± 0.02, *R*^S^ = 0.13 ± 0.04) (Fig. 1a,b and Extended Data Fig. 1). By contrast, when we inhibited cellulose biosynthesis and loosened the cell wall with ES20, ES20-1 or isoxaben treatments^17^, we observed a loss of autophagic puncta and a notable relocalization of mCherry–ATG8A to the tonoplast (ES20-1: *R*^P^ = 0.96 ± 0.04, *R*^S^ = 0.93 ± 0.05; isoxaben: *R*^P^ = 0.95 ± 0.03, *R*^S^ = 0.87 ± 0.11). Similarly, treatment with Driselase, a fungal enzymatic cocktail that is used to mimic fungal infection^12^, also led to the labelling of the tonoplast with mCherry–ATG8A (*R*^P^ = 0.92 ± 0.03, *R*^S^ = 0.91 ± 0.05) (Fig. 1a,b and Extended Data Fig. 1). Tonoplast labelling upon cell wall damage is not specific to the ATG8A isoform or an artefact of the mCherry tag, since all nine *Arabidopsis* GFP-labelled ATG8 isoforms showed similar tonoplast relocalization upon ES20-1 treatment (Extended Data Fig. 2a).

We next implemented ascorbate peroxidase 2 (APEX2)-based electron microscopy to study tonoplast ATG8ylation at the ultrastructural level. APEX2 is a plant peroxidase enzyme that has been engineered as a genetically encoded electron microscopy tag. The addition of the substrate diaminobenzidine (DAB) leads to localized reactive oxygen species production and increases contrast in electron micrographs^18^. Without DAB, there was minimal background labelling in *Arabidopsis* root samples (Fig. 1c). The addition of the substrate led to some background labelling at the Golgi apparatus, suggesting that inherent plant peroxidase activity could lead to the labelling of the Golgi apparatus (Extended Data Fig. 2b). Nevertheless, the substantial increase in the contrast prompted us to test the APEX2–ATG8A samples upon Torin and ES20-1 treatments. We could visualize typical autophagosome structures in Torin-treated samples (Fig. 1c). By contrast, upon ES20-1 treatment, we saw clear labelling of the tonoplast owing to APEX2 activity (Fig. 1c). In micrographs obtained from ES20-1-treated samples, we could also detect invaginations of the tonoplast that were densely labelled with APEX2–ATG8A (Fig. 1c). Altogether, consistent with our confocal microscopy results, APEX2-based electron microscopy showed relocalization of ATG8A to the tonoplast upon cell wall damage.

In canonical macroautophagy, ATG8 is conjugated to the growing double-membraned phagophore via a lipid modification. Through a ubiquitination-like reaction, the C-terminal glycine residue gets lipidated for membrane insertion^19^. To test whether ATG8 is conjugated to the tonoplast, we tested the relocation of the terminal glycine mutant ATG8A-G117A, which cannot get lipidated. GFP–ATG8A-G117A had a diffuse pattern and failed to relocate to the tonoplast upon ES20-1 treatment (Fig. 1d). Collectively, these findings suggest that cell wall damage induces CASM at the tonoplast.

Finally, we investigated the evolutionary conservation of this pathway by testing whether cell wall damage induces tonoplast ATG8ylation in the early-diverging land plant *M. polymorpha*. We labelled *Marchantia* GFP–MpATG8A and GFP–MpATG8B and showed that upon ES20-1 treatment both ATG8 isoforms relocate from puncta-like autophagosomes to the tonoplast (Fig. 1e and Extended Data Fig. 2c). These results suggest that cell-wall-damage-induced tonoplast ATG8ylation is conserved across land plants. Additionally, we found that alkaline stress induces the same phenotype (Extended Data Fig. 2d).

### Genetic basis of tonoplast ATG8ylation

**Fig. 2:**
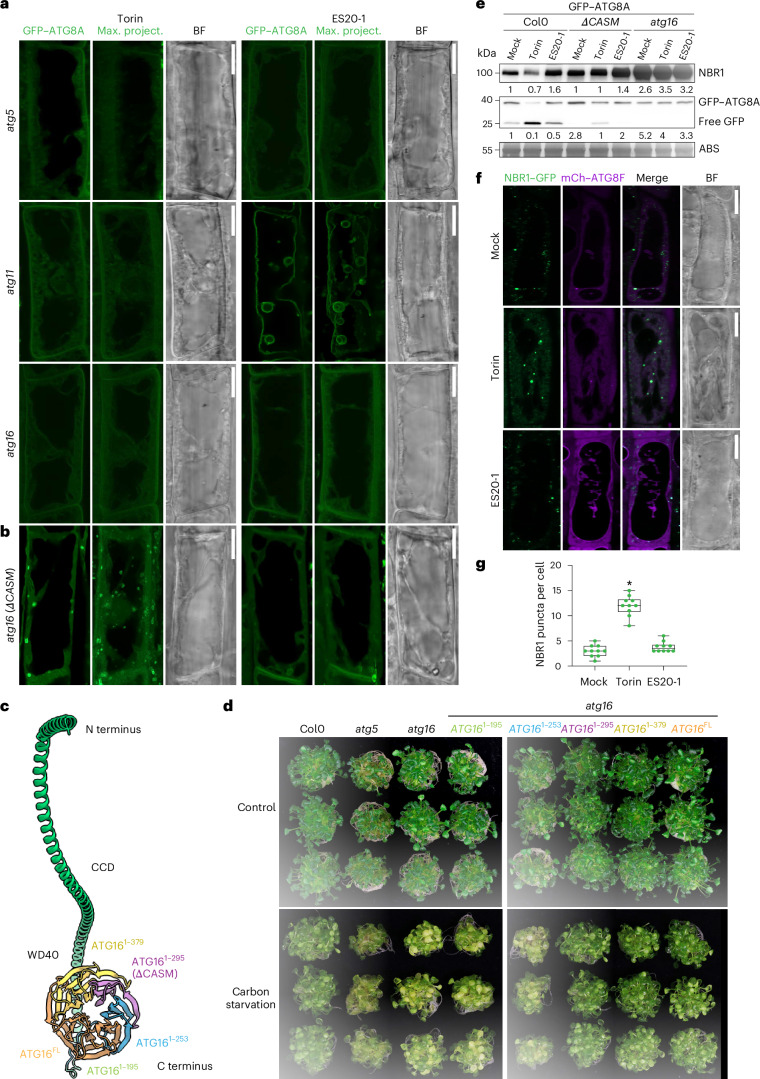
Genetic basis of tonoplast ATG8ylation. **a**, Confocal micrographs of GFP–ATG8A expressed in the *atg5*, *atg11* and *atg16* mutant backgrounds of *A. thaliana* root cells treated with Torin (1.5 h, 9 µM) or ES20-1 (8 h, 100 µM). A single optical slice, a maximum-intensity projection (20 µm depth) and a bright field image are shown. Representative images from ten seedlings were analysed under each treatment. Scale bars, 10 µm. **b**, Same setup as in **a** but showing the *atg16* mutant complemented with ΔCASM, which retains canonical autophagy but lacks non-canonical autophagy. Representative images from ten seedlings were analysed under each treatment. Scale bars, 10 µm. **c**, Schematic of ATG16 protein domains (coiled-coil domain (CCD) and WD40) showing the truncations used to complement atg16 mutants, with colours indicating the retained regions: 1–195 (green), 1–253 (blue), 1–295 (purple, ΔCASM), 1–379 (yellow) and full-length (FL) (orange). **d**, Carbon starvation assay across three replicates for wild-type (Col0), *atg5*, *atg16* and complemented lines. ΔCASM and longer variants resist carbon starvation, unlike *atg16* and other mutants. **e**, Western blot analysis of plant material expressing GFP–ATG8A in the Col0, *ΔCASM/atg16* and *atg16* backgrounds under mock, Torin (4 h, 9 µM) or ES20-1 (8 h, 100 µM) treatments. The blots were probed with anti-NBR1 and anti-GFP. Amido black staining (ABS) was used as the loading control. NBR1 intensity values are normalized to the loading control and presented as the average of three replicates. ATG8 intensity values are the ratio of GFP–ATG8A against free GFP and are the average of three replicates. **f**, Confocal micrographs showing NBR1–GFP localization (green) and mCh–ATG8F (magenta) in *Arabidopsis* root cells, under mock, Torin (1.5 h, 9 µM) and ES20-1 (8 h, 100 µM) treatments. The images include separate channels for NBR1–GFP and mCh–ATG8F, a merged image and a corresponding bright field image. Scale bars, 10 µm. **g**, Quantification of NBR1 puncta across treatments. One-sided Wilcoxon tests compared the treatments (*n* = 10) to mock; significant differences (*P* < 0.01) are indicated with asterisks. In each box plot, the central line indicates the median, and the upper and lower bounds represent quartile 3 (75th percentile) and quartile 1 (25th percentile), respectively. The whiskers denote the minima and maxima of the data points. Source data

**Extended Data Fig. 3:**
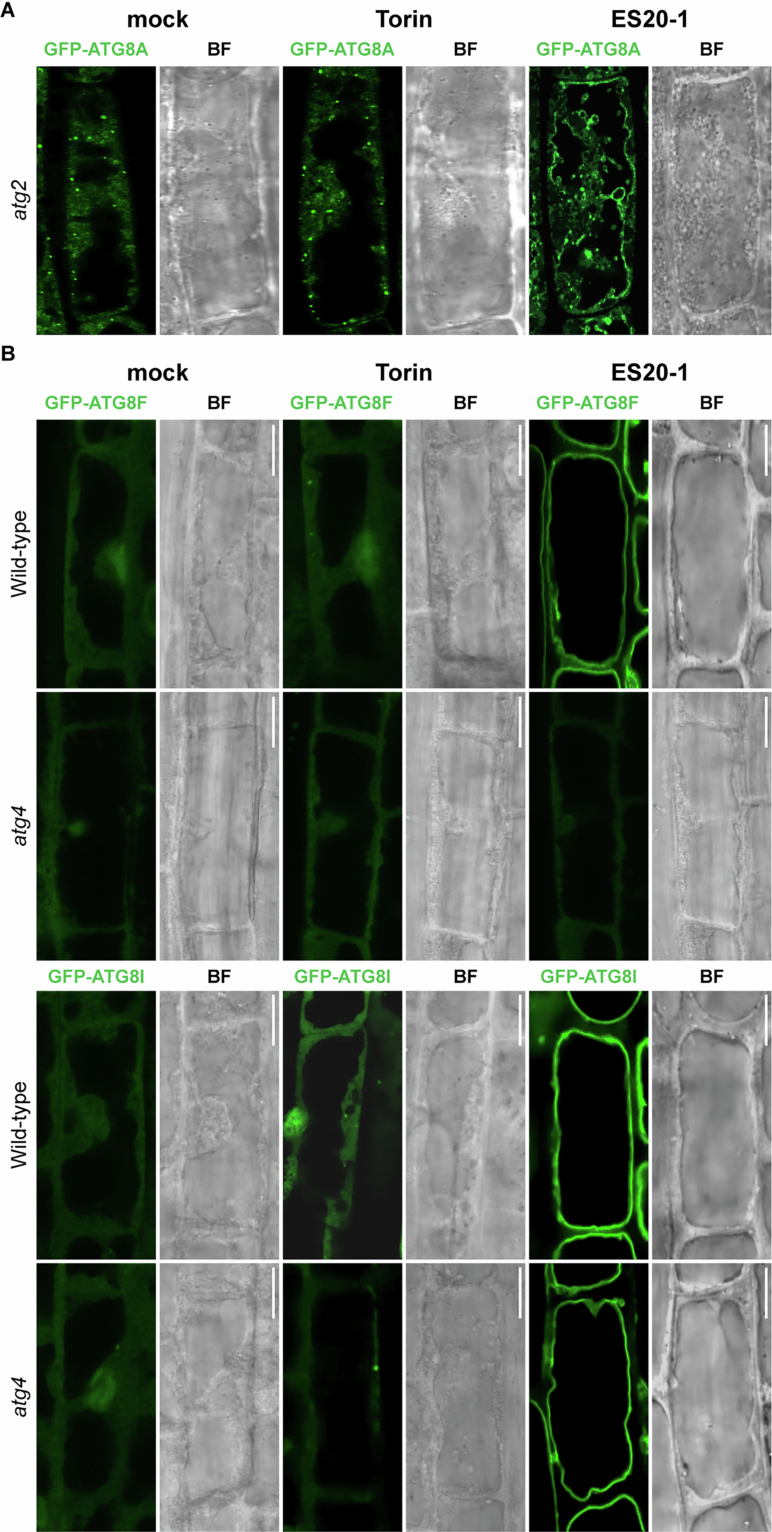
Genetic basis of tonoplast ATG8ylation. **(a)** Confocal micrographs of GFP-ATG8A expressed in *atg2* mutant background of *Arabidopsis thaliana* root cells treated with Torin (1.5 hours, 9 µM) or ES20-1 (8 hours, 100 µM). Each panel includes a single optical slice and a corresponding bright field image to illustrate ATG8A behavior. Representative image from 10 seedlings analyzed under each treatment. Scale bar, 10 µm. **(b)** Confocal micrographs of GFP-ATG8F or GFP-ATG8I expressed in wild-type or *atg4* mutant background of *Arabidopsis thaliana* root cells treated with Torin (1.5 hours, 9 µM) or ES20-1 (8 hours, 100 µM). Each panel includes a single optical slice and a corresponding bright field image to illustrate ATG8A behavior. Representative image from 10 seedlings analyzed under each treatment. Scale bar, 10 µm.

Next, we sought to dissect the genetic basis of tonoplast ATG8ylation. Previous studies in mammalian cells have shown that unlike autophagy, CASM requires only a subset of the ATG proteins^20^. We generated stable transgenic *Arabidopsis* lines that express GFP–ATG8A in the mutant background of the core ATG proteins ATG2, ATG5, ATG11 and ATG16. As expected, autophagosome formation is blocked in all four mutant backgrounds (Fig. 2a and Extended Data Fig. 3a). By contrast, tonoplast ATG8ylation is inhibited only in *atg5* and *atg16* mutants, while it is not affected in *atg11* mutants and only slightly affected in *atg2* mutants (Fig. 2a and Extended Data Fig. 3a). This suggests that tonoplast ATG8ylation requires the ATG8 conjugation machinery but is independent of the ATG1 kinase complex that initiates autophagosome formation. In the *atg2* mutant background, ATG8 decorates the tonoplast, though the signal is less uniform than that of the wild type (Extended Data Fig. 3a). The ATG2–ATG18 complex, which is involved in lipid transfer to the newly formed autophagosome^14^, may still contribute to this process but appears to be non-essential for tonoplast ATG8ylation. We also checked ATG8ylation in the *atg4* mutant background using two different ATG8 isoforms, GFP–ATG8F and GFP–ATG8I. ATG4 is a protease that cleaves ATG8 to expose the C-terminal glycine residue that gets lipidated^15^. Interestingly, in *Arabidopsis*, the ATG8I isoform naturally ends with a glycine residue and therefore does not require ATG4 processing^21^. Consistently, while ATG8F conjugation to the tonoplast was inhibited in the *atg4a/b* double mutant background, ATG8I conjugation was not affected (Extended Data Fig. 3b).

**Extended Data Fig. 4:**
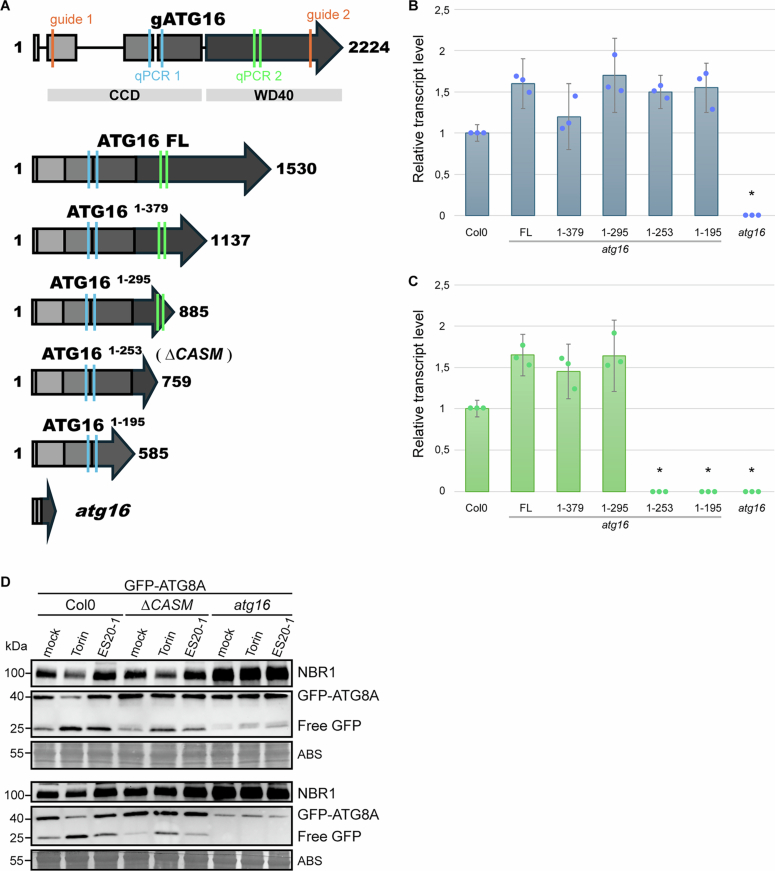
Generation of the atg16 mutant and the complementation lines. **(a)** Diagram showing a representation of the ATG16 gene, the *atg16* mutant generated by CRISPR and the different truncations used to complement the *atg16* phenotype. The position of the gRNAs for CRISPR (orange) and the primers for two qPCRs (blue – qPCR1- and green -qPCR2-) are indicated. **(b, c)** Bar graph showing the relative gene expression levels of ATG16 on different genotypes: Col0, *atg16* ATG16 FL, *atg16* ATG16^1-379^, *atg16* ATG16^1-295^, *atg16* ATG16^1-253^, *atg16* ATG16^1-195^ and *atg16*. Two different qPCR were performed, targeting the end of Exon 3 and beginning of Exon 4 **(b)** and targeting the middle of Exon 5 **(c)**. Gene expression is presented as fold changes normalized to the reference gene (ACT2) and calculated using the ΔΔCt method. Data are presented as mean values from three independent biological replicates, each with three technical replicates, plus SEM. Statistical significance was determined by a two-tailed Student’s t-test (p < 0.05) and represented with an asterisk. **(d)** Replicates of the western blot analysis of plant material expressing GFP-ATG8A in Col0, *ΔCASM/atg16*, and *atg16* backgrounds of Fig. 2e. Source data

Mammalian ATG16L1 has a key role in determining the site of LC3/ATG8 conjugation. During autophagy, the mammalian ATG16L1 coiled-coil domain interacts with the phosphatidylinositol 3-phosphate binding protein WIPI2 to conjugate LC3/ATG8 to the growing phagophore^22,23^. During CASM, the WD40 domain of mammalian ATG16L1 mediates ATG8 conjugation to single membranes^24^. Given the role of ATG16 in defining the site of ATG8 conjugation, we hypothesized that complementation of the *atg16* mutant with truncated ATG16 variants could provide a genetic tool whereby autophagy could still happen, but CASM is inhibited (Extended Data Fig. 4a–c). To test this, we complemented the *atg16* mutant with different C-terminal truncations of ATG16 where the WD40 domain is located and tested autophagic flux, carbon starvation sensitivity and autophagosome biogenesis (Fig. 2b–e). Complementation with ATG16^1–295^ (hereafter ΔCASM) restored canonical autophagy: (1) GFP–ATG8A formed punctate structures upon Torin treatment (Fig. 2b); (2) unlike the *atg16* or *atg5* mutants, complemented seedlings were insensitive to carbon starvation (Fig. 2d); and (3) there was an increase in free GFP and a decrease in the autophagy receptor NBR1 protein^25^ levels upon Torin treatment, indicative of functional autophagic flux (Fig. 2e and Extended Data Fig. 4d). By contrast, tonoplast ATG8ylation was inhibited in *ΔCASM* cells (Fig. 2b). These results suggest that the ATG16 WD40 domain is essential for CASM in *Arabidopsis*. Crucially, the *ΔCASM* line provides us a genetic tool that bypasses the pleiotropic effects seen with *atg* mutants, thus allowing for targeted investigation into the physiological and cellular significance of tonoplast ATG8ylation following cell wall damage.

**Extended Data Fig. 5:**
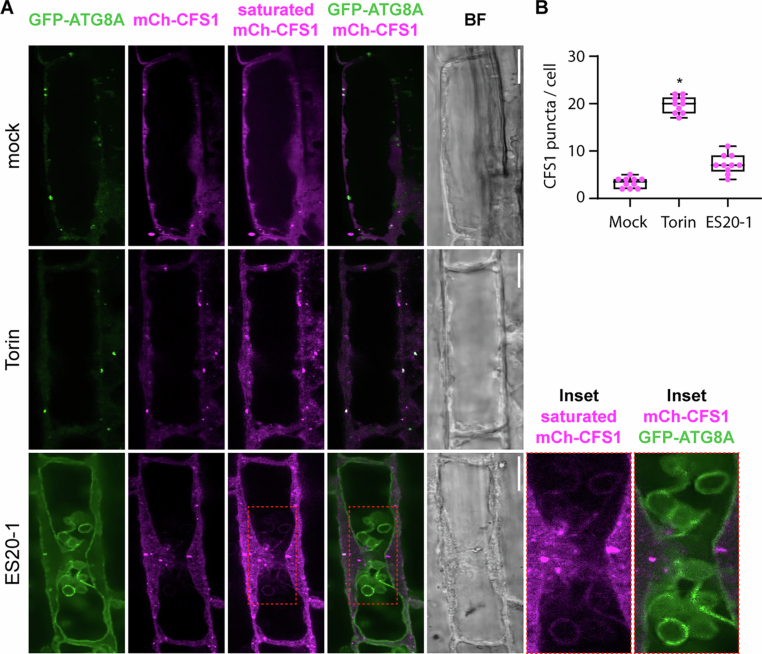
Autophagy adaptor CFS1 is not recruited to the tonoplast upon cell wall damage. **(a)** Confocal micrographs obtained from GFP-ATG8A mCh-CFS1 stably co-expressing *Arabidopsis thaliana* root cells under mock, Torin (1.5 hours, 9 µM), and ES20-1 (8 hours, 100 µM) treatments. The panel sequence includes single optical slices for GFP-ATG8A and mCh-CFS1, an additional panel for mCh-CFS1 with oversaturation to enhance visualization, a merge of both channels, corresponding bright field images and insets to enhance visualization. Scale bar, 10 µm. **(b)** Quantification of CFS1 puncta across the different treatment conditions used in (A). One-sided Wilcoxon test compared treatments (n = 10) to mock; significant differences (p < 0.01) are indicated as asterisks. The central line indicates the median, and the upper and lower bounds represent quartile 3 (75th percentile) and quartile 1 (25th percentile), respectively. The whiskers denote the minima and maxima of the data points.

We next tested whether all autophagic processes are rerouted to the tonoplast, by checking the localizations of the archetypical selective autophagy receptor NBR1 (ref. ^25^) and the recently characterized plant selective autophagy adaptor CFS1 (ref. ^26^). Both proteins interact with ATG8 directly via their conserved ATG8-interacting motifs^25,26^. NBR1 is located within the autophagosomes together with its cargo, whereas CFS1 is located on the outer autophagosome membrane and interacts with ESCRT machinery for autophagosome sorting^25,26^. Although both proteins responded to Torin treatment and formed more punctate structures, they did not relocate to the tonoplast upon ES20-1 treatment (Fig. 2f,g and Extended Data Fig. 5a,b). Notably, we observed some CFS1 vacuolar localization, although rather weak, upon ES20-1 treatment (Extended Data Fig. 5a,b). Since CFS1 is located on the outer autophagosome membrane, a blockage of the autophagic flux could lead to weak vacuolar localization of this protein. Nevertheless, these findings indicate that cell wall damage does not reroute all autophagic processes; rather, it triggers selective ATG8ylation of the tonoplast.

**Extended Data Fig. 6:**
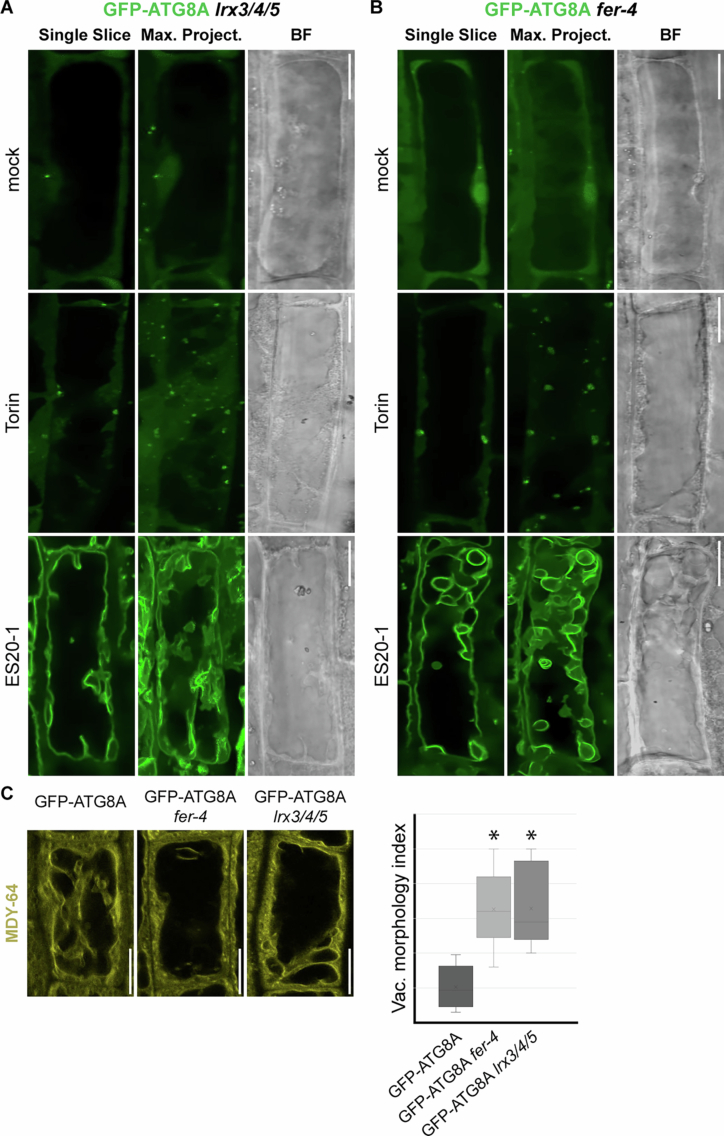
Tonoplast ATG8ylation does not require FERONIA. **(a)** Confocal micrographs of GFP-ATG8A expressed in *Arabidopsis thaliana lrx3/4/5* triple mutant background **(a)** or *fer4* mutant background **(b)**, treated with mock, Torin (1.5 hours, 9 µM), and ES20-1 (8 hours, 100 µM). The set includes a single optical slice, a maximum intensity projection, and a bright field image for each treatment condition. Representative image from 10 seedlings analyzed under each treatment. Scale bar, 10 µm. **(c)** Representative images and quantification of vacuolar morphology of late meristematic atrichoblast cells of GFP-ATG8A, GFP-ATG8A *fer-4* and GFP-ATG8A *lrx3/4/5*. MDY‐64 (yellow) staining depicts vacuolar membrane. One-sided Wilcoxon test compared treatments (n = 10) to GFP-ATG8A line; significant differences (p < 0.01) are indicated as asterisks. Scale bar, 10 µm. The central line indicates the median, and the upper and lower bounds represent quartile 3 (75th percentile) and quartile 1 (25th percentile), respectively. The whiskers denote the minima and maxima of the data points. Mean values are represented with ‘x’.

Since the CWI sensor FERONIA and its interacting partners LRX (leucine rich repeat extensin) family proteins were previously shown to link CWI to vacuolar morphology^9^, we next tested whether FERONIA regulates tonoplast ATG8ylation. We expressed GFP–ATG8A in *fer-4* and *lrx3/4/5* mutants. GFP–ATG8A localization after Torin or ES20-1 treatment was indistinguishable from that in the wild-type Columbia (Col0) background in both mutant backgrounds (Extended Data Fig. 6a,b), indicating that the FERONIA signalling pathway does not function in tonoplast ATG8ylation. Mutant backgrounds were checked for their known vacuolar morphology phenotype for validation (Extended Data Fig. 6c).

Together, these findings demonstrate that CASM and canonical autophagy are two independent pathways that are regulated by different protein networks.

### The role of tonoplast ATG8ylation

**Fig. 3:**
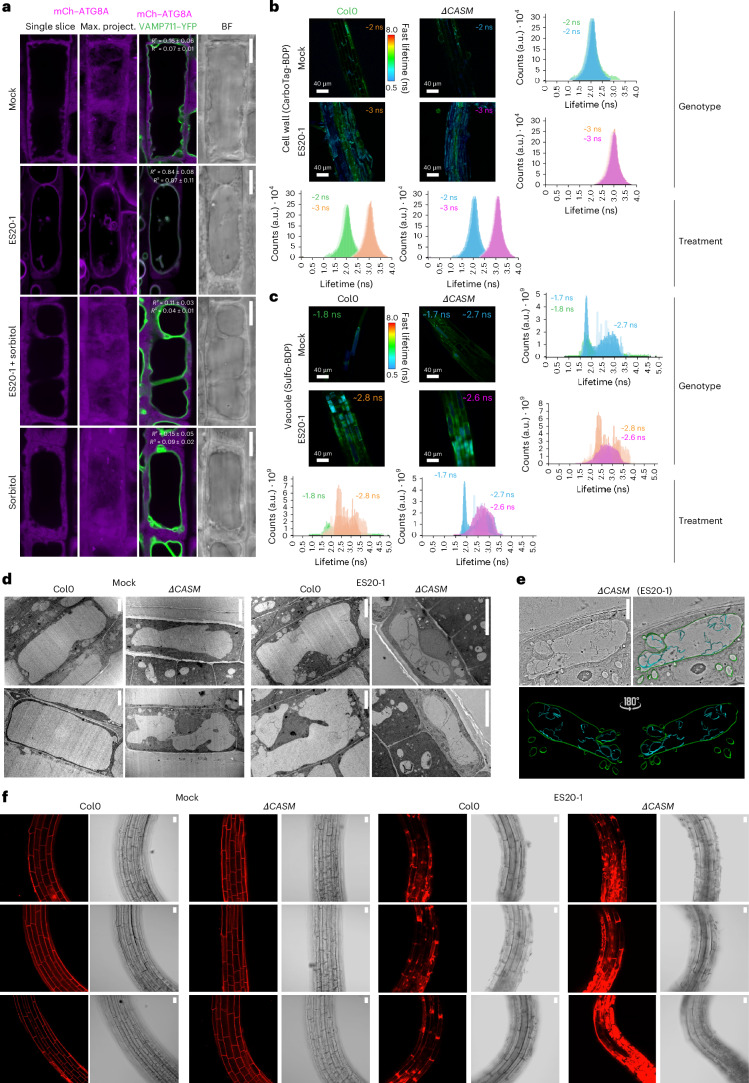
Tonoplast ATG8ylation maintains vacuolar integrity upon cell wall damage in a turgor-pressure-dependent manner. **a**, Confocal micrographs of *Arabidopsis* root cells expressing mCh–ATG8A and VAMP711–YFP, highlighting ATG8A localization and tonoplast integrity under mock, ES20-1 (8 h, 100 µM), sorbitol (8 h, 50 mM) and combined sorbitol (8 h, 50 mM) + ES20-1 (8 h, 100 µM) treatments. A single slice of mCh–ATG8A, a maximum projection, a merged image with VAMP711–YFP and a corresponding bright field image are shown. Pearson and Spearman colocalization analyses are presented to quantify colocalization under each condition. Scale bars, 10 µm. **b**, Fluorescence lifetime imaging microscopy (FLIM) analysis of the Col0 and *ΔCASM* backgrounds treated with CarboTag-BDP, a fluorescent cell wall mechanoprobe, for 30 min at 10 µM concentration, following mock or ES20-1 (8 h, 100 µM) treatments. The fluorescence lifetime of the probe across three biological replicates is shown, with average lifetimes reported in nanoseconds. Four comparative graphs detail the lifetime variance for each treatment and genotype. Scale bars, 40 µm. **c**, FLIM analysis using Sulfo-BDP, a vacuolar mechanoprobe, to assess vacuolar crowding under the same conditions. Lifetime measurements in nanoseconds highlight differences in vacuolar crowding between treatments and genetic backgrounds. Average lifetime was measured for all values below 2 ns (peak 1) and above 2 ns (peak 2). Scale bars, 40 µm. **d**, Transmission electron micrographs demonstrating vacuolar morphology changes in the Col0 and *ΔCASM* backgrounds under mock and ES20-1 (8 h, 100 µM) treatments. The images reveal the significant fragmentation and invaginations upon cell wall damage in the *ΔCASM* line. Scale bars, 5 µm. **e**, Electron tomography analysis of a *ΔCASM* root cell treated with ES20-1 (8 h, 100 µM), providing a detailed 3D visualization of vacuolar morphology and the surrounding cellular environment. The tomogram is presented with a 180° rotation to enhance structural observation. Scale bar, 5 µm. **f**, PI staining of root cells from the Col0 and *ΔCASM* backgrounds under mock and ES20-1 (8 h, 100 µM) treatments, assessing cell viability and membrane integrity. Three replicates are shown for each genotype and treatment. Scale bars, 10 µm.

Our main hypothesis is that cell wall damage will weaken the counterbalance that the cell wall provides against the turgor pressure contained within the vacuole and threaten vacuolar integrity. We first tested this hypothesis by combining ES20-1 treatments with osmolyte sorbitol treatment. Consistent with our hypothesis, sorbitol treatment, which lowers turgor pressure, suppressed tonoplast ATG8ylation upon ES20-1 treatment (Fig. 3a).

**Extended Data Fig. 7:**
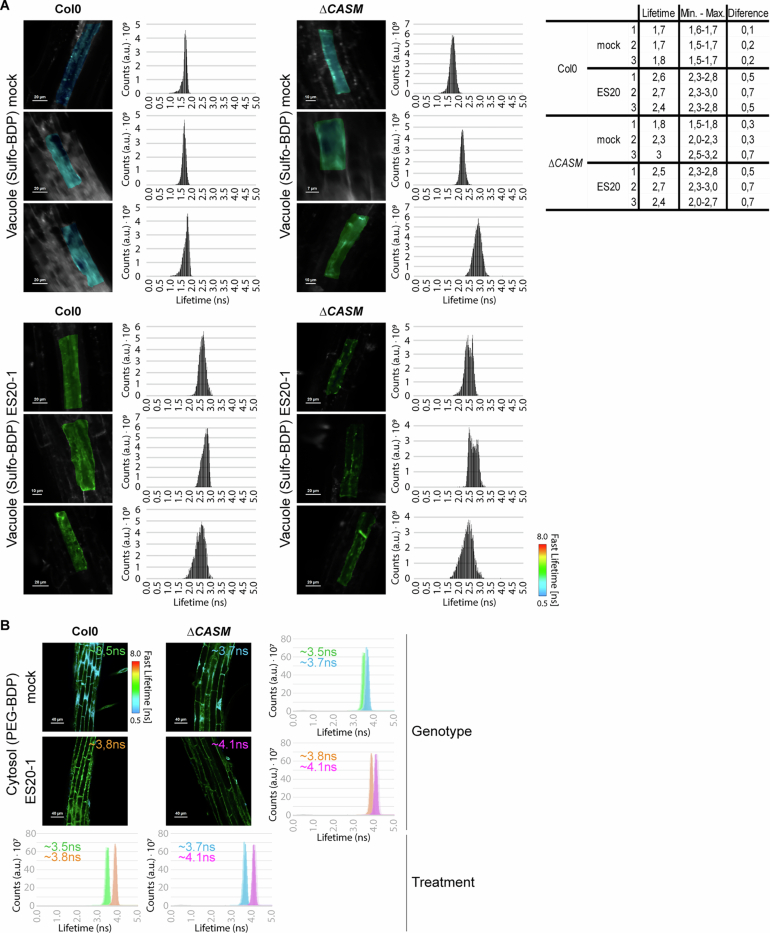
Cell wall damage affects vacuolar and cytoplasmic crowding. **(a)** FLIM analysis of Sulfo-BDP mechanoprobe to assess vacuolar crowding in single cells. Analysis of Col0 and *ΔCASM* backgrounds treated with Sulfo-BDP, a fluorescent vacuolar mechanoprobe, for 90 minutes at 10 µM concentration, following mock or ES20-1 (8 hours, 100 µM) treatments. The fluorescence lifetime of the probe across three biological replicates, with average lifetimes reported in nanoseconds. Average lifetime, minimum and maximum lifetime with over 2·10^9^ counts, and the subtraction of this maximum and minimum are provided in a supplemental table. Scale bar, 20 µm. **(b)** FLIM analysis of PEG-BDP mechanoprobe to assess cytoplasmic crowding. Analysis of Col0 and *ΔCASM* backgrounds treated with PEG-BDP, a fluorescent cytoplasmic mechanoprobe, for 30 minutes at 10 µM concentration, following mock or ES20-1 (8 hours, 100 µM) treatments. The fluorescence lifetime of the probe across three biological replicates, with average lifetimes reported in nanoseconds. Four comparative graphs detail the lifetime variance per treatment and genotype. Scale bar, 40 µm.

We then sought to determine whether cell wall damage affects cellular mechanics by taking advantage of the recently established cell wall, vacuole and cytoplasm targeted BODIPY-based mechanoprobes^27,28^. First, we measured the fluorescence lifetime of the cell wall porosity reporter CarboTag-BDP upon ES20-1 treatment in the wild-type Col0 and *ΔCASM* lines. The CarboTag-BDP probe had increased lifetime in both Col0 and *ΔCASM* seedlings (mock, ~2 ns; ES20-1, ~3 ns), which indicates decreased cell wall porosity and demonstrates cell wall defects caused by ES20-1 treatment (Fig. 3b). We then tested the fluorescence lifetime of the vacuolar mechanoprobe Sulfo-BDP to test molecular crowding inside the vacuole. Sulfo-BDP showed two peaks in our lifetime measurements (one peak below 2 ns and a second peak above 2 ns). *ΔCASM* seedlings already had vacuoles emitting higher lifetime signals under control conditions (first peak: Col0, ~1.8 ns; *ΔCASM*, ~1.7 ns; second peak: Col0, none; *ΔCASM*, ~2.7 ns), suggesting that the vacuoles are already damaged in *ΔCASM* cells (Fig. 3c). Upon ES20-1 treatment, all vacuoles had increased lifetimes in both genotypes tested (second peak: Col0, ~2.8 ns; *ΔCASM*, ~2.6 ns), further demonstrating the vacuolar damage triggered by cell wall damage (Fig. 3c). We realized that *ΔCASM* and ES20-1-treated samples had a wider distribution in their histograms than Col0 under mock conditions. So, we recorded single cells treated with Sulfo-BDP to check whether these wider histograms were due to differences inside the vacuole or due to different vacuoles having different viscosity (Extended Data Fig. 7a). In Col0, the viscosity inside the vacuole is tightly controlled. Vacuole lumens are homogeneous (narrow peaks) and very similar between different cells. After ES20-1 treatment the average lifetime is higher, and the peaks are wider. In the *ΔCASM* mutant background, the vacuoles themselves are more heterogeneous, and there is also more variation between cells. This is suggestive of issues in maintaining vacuolar integrity. After ES20-1 treatment all vacuoles look damaged, leading to a homogeneous distribution of lifetime measurements. Altogether, these results suggest that ES20-1 treatment causes cell wall damage and impedes vacuolar integrity.

Finally, we tested the cytoplasmic mechanoprobe PEG-BDP upon ES20-1 treatment. Both Col0 and *ΔCASM* had increased lifetimes (mock: Col0, ~3.5 ns; *ΔCASM*, ~3.7 ns; ES20-1: Col0, ~3.8 ns; *ΔCASM*, ~4.1 ns) (Extended Data Fig. 7b). However, the PEG-BDP fluorescence lifetime was consistently higher in *ΔCASM* than in Col0, suggesting that cytosolic viscosity is more severely affected in cells that lack tonoplast ATG8ylation, which is indicative of defects in turgor mechanostasis due to vacuolar defects (Extended Data Fig. 7b). In-depth studies are necessary to understand the molecular basis of the increased cytosolic viscosity in the *ΔCASM* line, but these findings prompted us to further investigate vacuolar integrity upon cell wall damage.

**Extended Data Fig. 8:**
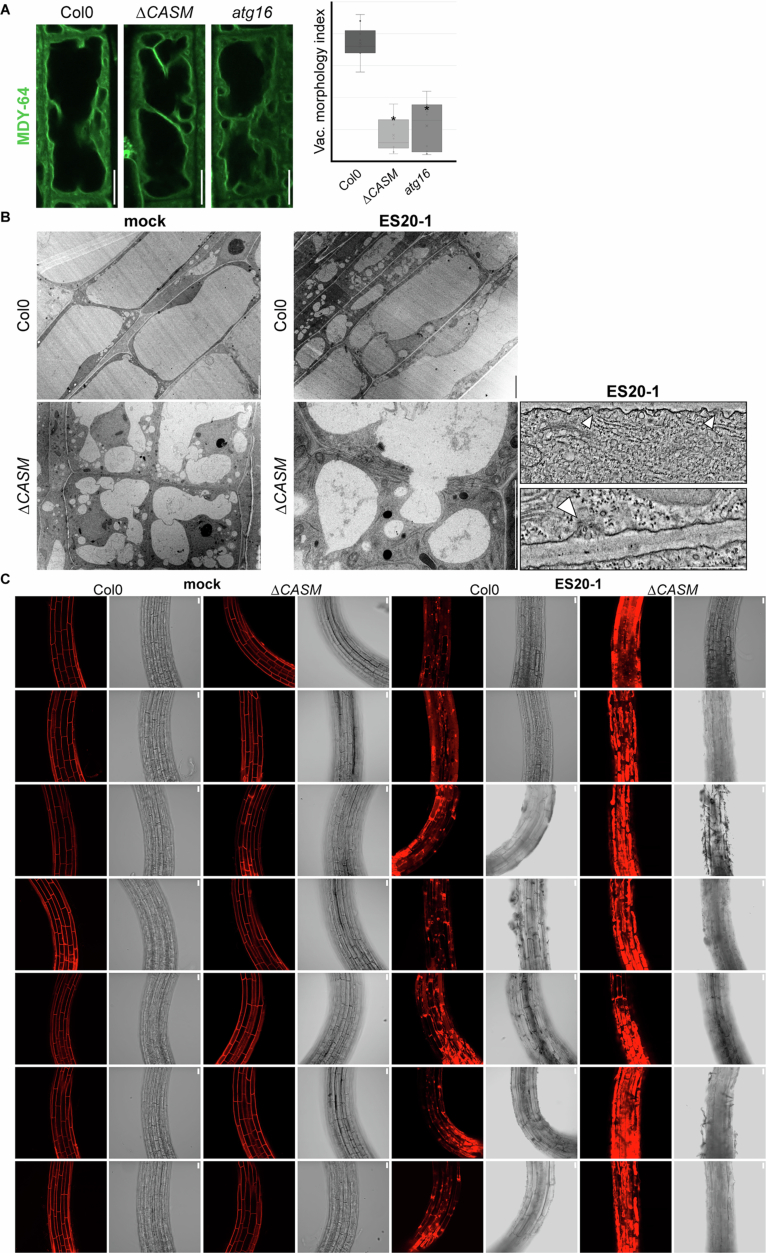
Tonoplast ATG8ylation contributes to vacuolar and cellular homeostasis upon cell wall damage. **(a)** Vacuolar morphology analyses upon cell wall damage. Representative images and quantification of vacuolar morphology of Col0, *ΔCASM* and *atg16* root cells from the early elongation zone. MDY‐64 (green) staining depicts vacuolar membrane. One-sided Wilcoxon test compared treatments (n = 10) to Col0 wild-type; significant differences (p < 0.01) are indicated as asterisks. The central line indicates the median, and the upper and lower bounds represent quartile 3 (75th percentile) and quartile 1 (25th percentile), respectively. The whiskers denote the minima and maxima of the data points. Scale bar, 10. **(b)** Transmission Electron Microscopy (EM) images visualizing the vacuole in Col0 and *ΔCASM* backgrounds under mock and ES20-1 (8 hours, 100 µM) treatments. The images reveal vacuolar fusion between cells in response to cell wall damage. Insets of cell wall damage upon ES20-1 treatment are also included. Representative image from 3 seedlings analyzed under each treatment. Scale bar, 5 µm for the zoom out images and 0,5 µm for the insets of the cell wall. **(c)** *ΔCASM* root cells are more sensitive to cell wall damage. Propidium Iodide (PI) staining of root cells from Col0 and *ΔCASM* backgrounds under mock and ES20-1 (8 hours, 100 µM) treatments, assessing cell viability and membrane integrity. Seven more replicates of Fig. 3f are shown for each genotype and treatment. Scale bar, 10 µm.

To further assess vacuolar integrity, we visualized vacuolar morphology using transmission electron microscopy and electron tomography. Compared with the mock condition, ES20-1 treatment induced vacuolar invaginations in the wild-type Col0 background (Fig. 3d). The *ΔCASM* mutant already showed altered vacuolar morphology and invaginations under control conditions, which became more severe upon ES20-1 treatment (Fig. 3d,e, Extended Data Fig. 8a and Supplementary Video 1). On some occasions, we even observed vacuoles traversing to the adjacent cells through the holes formed upon ES20-1 treatment (Extended Data Fig. 8b).

Finally, we tested the contribution of tonoplast ATG8ylation to cellular integrity with propidium iodide (PI) staining. PI cannot permeate living cells and is commonly used as a cell viability assay^12,29^. Upon ES20-1 treatment, we observed an increase in PI staining, indicative of increased cell death, in both Col0 and the *ΔCASM* mutant (Fig. 3f and Extended Data Fig. 8c). However, cell death after ES20-1 treatment was significantly exacerbated in the *ΔCASM* mutant. Altogether, these findings demonstrate that tonoplast ATG8ylation is essential to maintaining vacuolar integrity and cell survival upon cell wall damage.

### Molecular basis of tonoplast ATG8ylation

**Extended Data Fig. 9:**
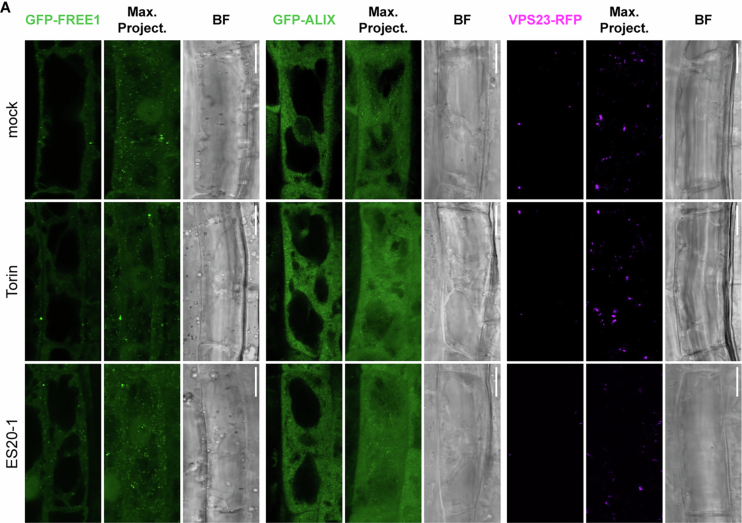
Key ESCRT proteins are not recruited to the tonoplast upon cell wall damage. Confocal micrographs depicting the localization of ESCRT (Endosomal Sorting Complex Required for Transport) machinery components GFP-FREE1, GFP-ALIX, and VPS23-RFP in *Arabidopsis thaliana* root cells, under mock, Torin (1.5 hours, 9 µM), and ES20-1 (8 hours, 100 µM) treatments. Each set comprises a single optical slice, a maximum intensity projection, and a corresponding bright field image, facilitating the comparative analysis of ESCRT component dynamics under torin and ES20-1 treatments. Representative image from 10 seedlings analyzed under each treatment. Scale bar, 10 µm.

Next, we sought to determine the molecular players involved in tonoplast ATG8ylation. Membrane repair is typically coordinated by the ESCRT complex^30–33^. To investigate a possible link between ESCRT and cell-wall-damage-induced tonoplast ATG8ylation, we visualized the key ESCRT proteins FREE1, ALIX and VPS23 upon ES20-1 treatment^34–36^. We observed no significant alterations in the localization of these proteins (Extended Data Fig. 9), suggesting that the ESCRT machinery is not involved in tonoplast ATG8ylation triggered by cell wall damage.

**Fig. 4:**
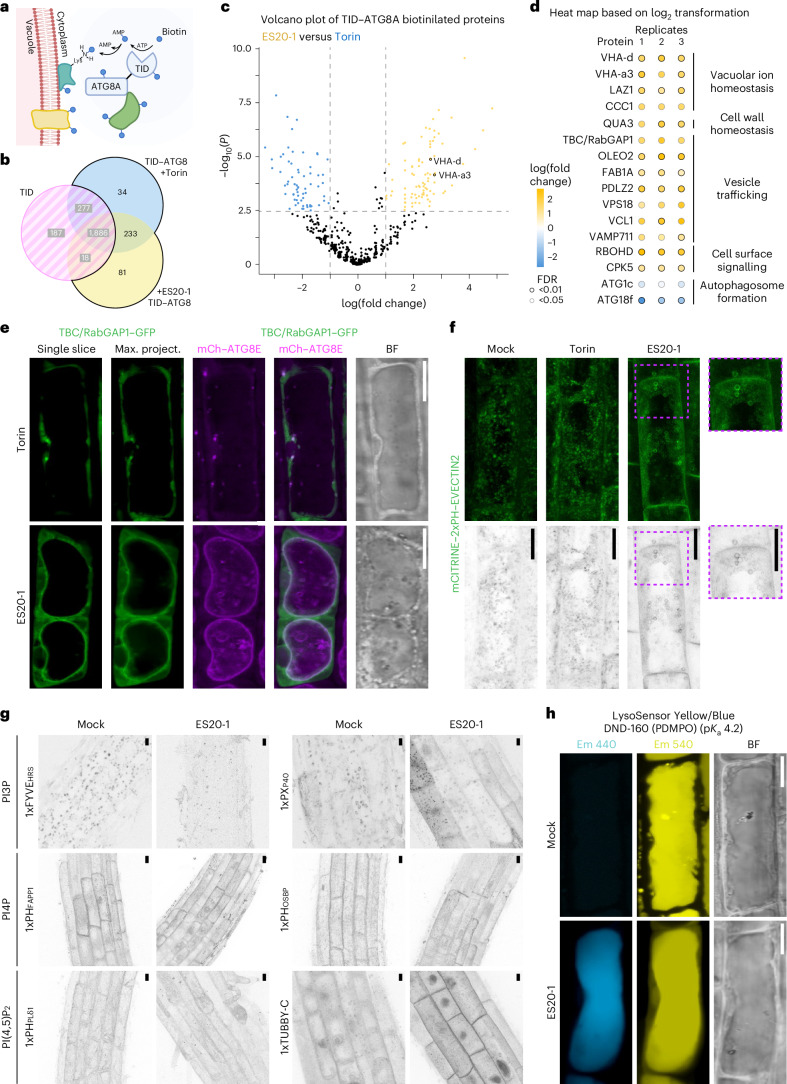
Molecular basis of tonoplast ATG8ylation. **a**, Proximity-dependent biotin labelling of ATG8A-interacting proteins during cell-wall-damage-induced tonoplast ATG8ylation. TID, TurboID. **b**, Venn diagram summarizing the results of TurboID-based proximity-labelling proteomics of TurboID–ATG8A under Torin (4 hs, 9 µM) and ES20-1 (8 h, 100 µM) treatments, highlighting overlap and unique proteins. TurboID alone is used as a negative control. **c**, Volcano plot of TurboID–ATG8A ES20-1 treatment versus Torin (*n* = 3 replicates, 348 total proteins). The yellow dots indicate proteins enriched in ES20-1 (Student’s *t*-test, *P* < 0.05, *n* = 81); the blue dots indicate those enriched in Torin (*P* < 0.05, *n* = 34). **d**, Proteins in five categories shown as dot plots. Circle colour represents log fold change; edge colour indicates confidence (Student’s *t*-test with false discovery rate (FDR) correction, FDR < 0.05, grey; FDR < 0.01, black). **e**, Confocal micrographs showing the localization of TBC/RabGAP1–GFP and mCh–ATG8E in *A. thaliana* root cells, under mock or ES20-1 (8 h, 100 µM) treatments. A single optical slice, a maximum projection, a merged image and a bright field image are shown. Representative images from ten seedlings were analysed under each treatment. Scale bars, 10 µm. **f**, PS biosensor mCITRINE–2xPH–EVECTIN2 changes localization upon cell wall damage. Confocal micrographs of *A. thaliana* root cells under mock, Torin (1.5 h, 9 µM) and ES20-1 (8 h, 100 µM) treatments are shown. Maximum-intensity projections are shown in green and inverted greyscale. Representative images from ten seedlings were analysed under each treatment. Scale bars, 10 µm. **g**, Confocal micrographs depicting the localization of six different PIP sensors in *A. thaliana* root cells, under mock and ES20-1 (8 h, 100 µM) treatments. 1xFYVE^HRS^ and 1xPX^P40^ target phosphatidylinositol 3-phosphate (PI3P), 1xPH^FAPP1^ and 1xPH^OSBP^ target phosphatidylinositol 4-phosphate (PI4P) and 1xPH^PLδ1^ and 1xTUBBY-C target phosphatidylinositol 4,5-bisphosphate (PI(4,5)P_2_). All images are represented as inverted greyscale. Representative images from ten seedlings were analysed under each treatment. Scale bars, 10 µm. **h**, Cell wall damage increases vacuolar pH. Confocal images of Col0 roots treated with LysoSensor Yellow/Blue DND-160 under mock or ES20-1 (8 h, 100 µM) conditions are presented, showing dual emission (Em) in yellow and blue on the basis of pH. Representative images from ten seedlings were analysed under each treatment. Scale bars, 10 µm.

**Extended Data Fig. 10:**
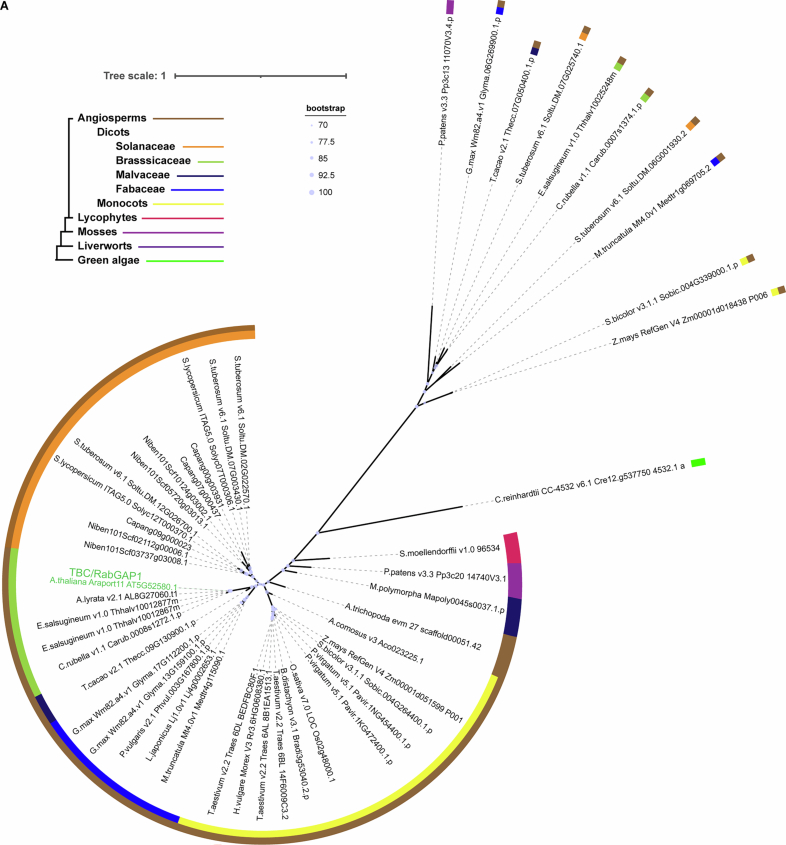
TBC/RabGAP1 is conserved in plants. A plant phylogeny displaying the presence of TBC/RabGAP1 across diverse species. Major plant taxonomic groups are denoted with a colored ribbon. Bootstrap confidence above 70 is shown at the bottom of each new leaf.

To identify proteins that regulate tonoplast ATG8ylation, we performed proximity-labelling proteomics of a TurboID-tagged ATG8A line upon Torin and ES20-1 treatments (Fig. 4a–d and Supplementary Table 1). We used TurboID alone as a negative control to deduct non-ATG8A-specific interactors and Torin treatment to remove general autophagy regulators. Consistent with our genetics findings, ATG1c and ATG18f were downregulated in ES20-1-treated samples, compared with Torin samples (Fig. 4d and Supplementary Table 1). The ATG8 proxitome upon ES20-1 treatment revealed several specific proteins that could be grouped into vacuolar ion homeostasis, vesicle trafficking, cell wall homeostasis and cell surface signalling (Fig. 4d). One of the ES20-1-specific interactors was a TBC/RabGAP protein, a homologue of which was recently shown to coordinate lysosomal repair upon damage in mammalian cells^37^. Phylogenetic analysis has shown that TBC/RabGAP1 is a singleton in *A. thaliana* (Extended Data Fig. 10). To test whether it is recruited to the tonoplast upon cell wall damage, we generated stable lines that co-express TBC/RabGAP1 and ATG8. Unlike NBR1 or ESCRT proteins, TBC/RabGAP1 is recruited to the tonoplast upon cell wall damage (Fig. 4e). Further studies are necessary to functionally characterize the role of TBC/RabGAP1, but these findings demonstrate that the ES20-1 proxitome is a useful resource to identify the regulators of tonoplast ATG8ylation.

Since we found many candidates involved in lipid trafficking and previous studies have suggested lipid transfer as an important part of lysosomal repair, we decided to check the localizations of phosphoinositide biosensors that mark phosphatidylinositol 3-phosphate, phosphatidylinositol 4-phosphate, phosphatidylinositol 4,5-bisphosphate and phosphatidylserine (PS) upon cell wall damage^38,39^. We found that only the PS biosensor changes localization after ES20-1 treatment (Fig. 4f). Under mock and Torin treatments, PS is localized to small vesicles distributed throughout the cytoplasm. Upon ES20-1 treatment, PS localizes to larger vesicles, resembling intravacuolar vesicles that we observe during CASM (Fig. 4g). Consistently, PS has been shown to be involved in CASM in mammalian cells^20^.

**Fig. 5:**
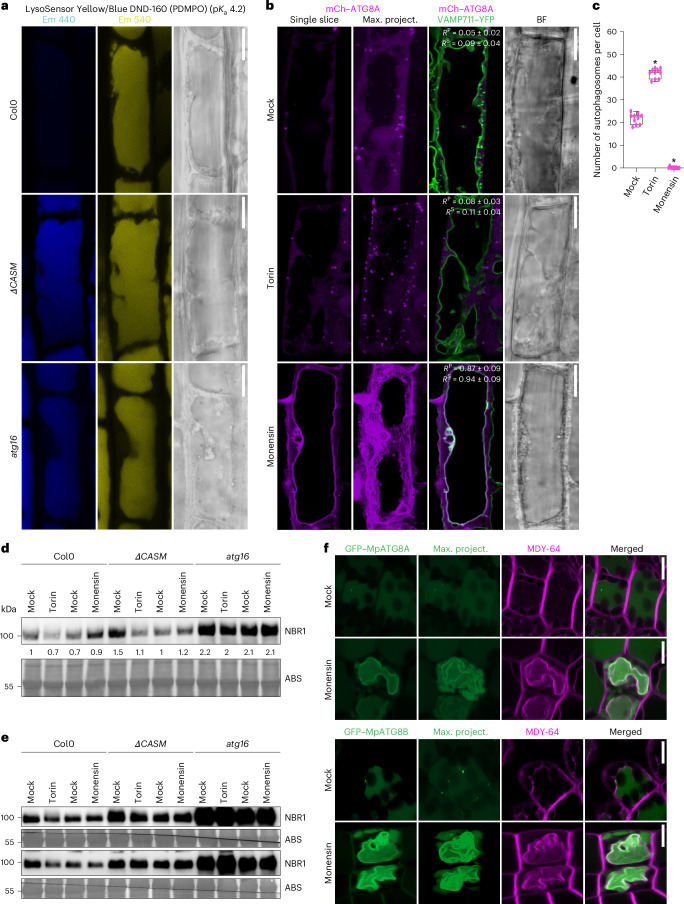
Vacuolar ionophore monensin changes vacuolar pH and triggers tonoplast ATG8ylation. **a**, Confocal images of Col0, *ΔCASM* and *atg16* roots treated with LysoSensor Yellow/Blue DND-160 under mock or ES20-1 (8 h, 100 µM) conditions, showing dual emission in yellow and blue on the basis of pH. Representative images from ten seedlings were analysed under each treatment. Scale bars, 10 µm. **b**, Confocal micrographs of root cells in the early elongation zone of *A. thaliana*, highlighting the localization of mCh–ATG8A (magenta) illustrating tonoplast ATG8ylation. The panel includes a single optical slice and a maximum-intensity projection of a whole cell (20 µm depth), alongside a merged image with VAMP711–YFP (tonoplast marker) and a corresponding bright field image. Scale bars, 10 µm. Pearson and Spearman colocalization values are presented, showing the association between ATG8A and the tonoplast. The treatment conditions include mock, Torin (1.5 h, 9 µM) and monensin (0.5 h, 200 µM) treatments. **c**, Quantification of autophagosomes under the treatment conditions depicted in **b**. One-sided Wilcoxon tests compared the treatments (*n* = 10) to mock; significant differences (*P* < 0.01) are indicated with asterisks. In each box plot, the central line indicates the median, and the upper and lower bounds represent quartile 3 (75th percentile) and quartile 1 (25th percentile), respectively. The whiskers denote the minima and maxima of the data points. **d**, Western blot analysis of NBR1 flux under mock, Torin (4 h, 9 µM) and monensin (0.5 h, 200 µM) treatments. NBR1 intensity values are normalized to the loading control and presented as the average of three replicates. **e**, Two replicates of the western blot in **d**. **f**, Confocal micrographs of GFP–MpATG8A and GFP–MpATG8B expressing *M. polymorpha* cells under mock or monensin (0.5 hs, 200 µM) treatments. MDY-64 (1 h, 1 µM) staining was used to mark tonoplast localization. Representative images from ten gemmae were analysed under each treatment. Scale bars, 10 µm. Source data

**Fig. 6:**
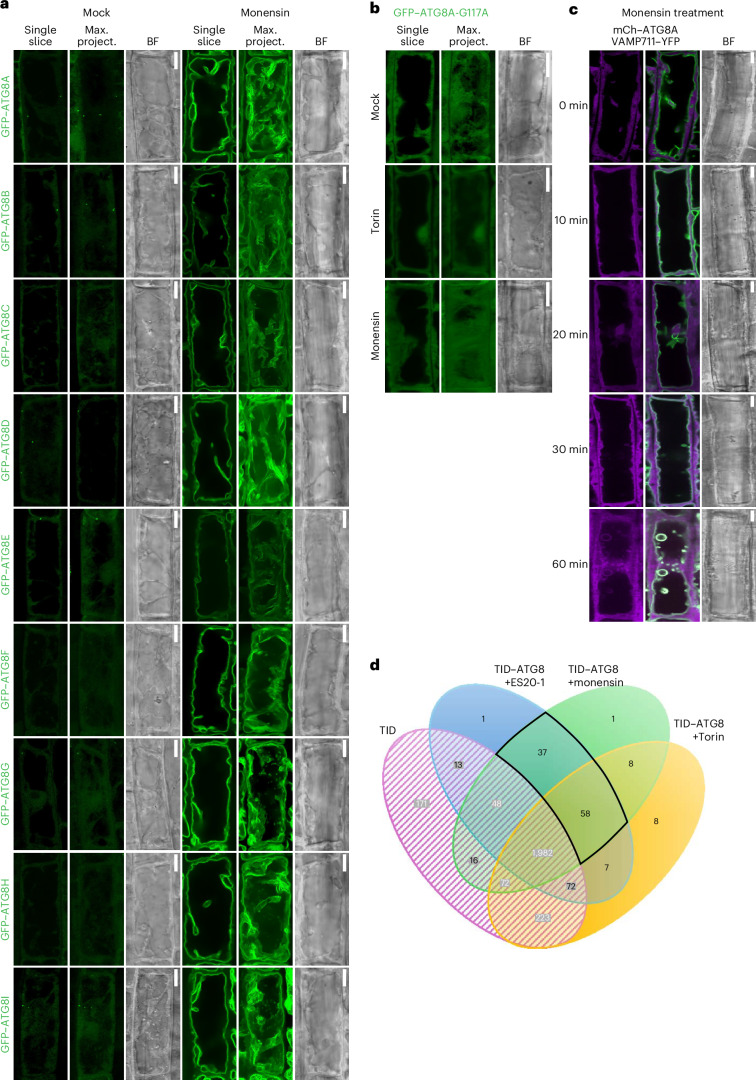
Vacuolar ionophore monensin triggers tonoplast ATG8ylation. **a**, Confocal micrographs displaying the localization of all nine GFP-tagged ATG8 isoforms (ATG8A to ATG8I) of *A. thaliana* under monensin (0.5 h, 200 µM) treatment. Representative images from ten seedlings were analysed under each treatment. Scale bars, 10 µm. **b**, Confocal micrographs of the GFP–ATG8A-G117A mutant, highlighting its localization in response to monensin (0.5 h, 200 µM) treatment. The images include single optical slices and maximum intensity projections. Representative images from ten seedlings were analysed under each treatment. Scale bars, 10 µm. **c**, Confocal micrographs of mCh–ATG8A (magenta) colocalized with the tonoplast marker VAMP711–YFP illustrating the recruitment of ATG8 to the tonoplast upon monensin treatment. The panel includes a single optical slice alongside a merged image with VAMP711–YFP and a corresponding bright field image. The images follow a time course treatment (0, 10, 20, 30 and 60 min) with monensin (200 µM). Representative images from ten seedlings were analysed under each treatment. Scale bars, 10 µm. **d**, Venn diagram summarizing the results of a TurboID-based proximity-labelling proteomics experiment with TurboID–ATG8A under Torin (1.5 h, 9 µM), ES20-1 (8 h, 100 µM) and monensin (2 h, 200 µM) treatments, highlighting the overlap and unique proteins identified across conditions. TurboID alone is used as a negative control.

Another group of proteins that caught our attention were V-ATPase subunits, VHA-a3 and VHA-d (Fig. 4c,d). V-ATPase is the primary proton pump at the vacuole that acidifies the vacuolar lumen and regulates a wide range of vacuolar functions, including CASM at the lysosomes^7,20,40^. To test whether V-ATPase also regulates tonoplast ATG8ylation, we first tested vacuolar pH upon cell wall damage. We measured the vacuolar pH upon ES20-1 treatment using the LysoSensor probe, which emits a fluorescence signal at 440 nm (blue) in compromised vacuoles^41^. We saw an increase in blue fluorescence, indicating that ES20-1 treatment increases the vacuolar pH (Fig. 4h). To further link vacuolar morphology changes to the pH of the vacuole, we checked for changes in the vacuolar pH in the *ΔCASM* and *atg16* mutant backgrounds. Consistent with a vacuolar homeostasis defect, in these mutants we observed higher pH than in the Col0 wild type (Fig. 5a). To directly test whether the assembly of V-ATPase is the driver of tonoplast ATG8ylation, we used the ionophore monensin, which serves as a proton–sodium antiporter and increases vacuolar pH, thereby promoting the assembly of V-ATPase^42^. Monensin treatment mimicked cell wall damage and (1) induced tonoplast ATG8ylation (Fig. 5b), (2) reduced autophagic puncta (Fig. 5c) (3) and blocked autophagic flux of NBR1 (Fig. 5d,e). Monensin-induced tonoplast ATG8ylation is also conserved in *Marchantia* (Fig. 5f). Similar to ES20-1 treatment, monensin induced the conjugation of all nine *Arabidopsis* ATG8 isoforms to the tonoplast (Fig. 6a,b). A time-course analysis of GFP–ATG8A upon monensin treatment revealed that within ~20 min, ATG8 is conjugated to the tonoplast, which is followed by fragmentation of the tonoplast at ~60 min (Fig. 6c). This aligns with recent findings that demonstrate the ability of ATG8 to alter the morphology of the membranes it attaches to^43^, suggesting that ATG8-mediated restructuring of the tonoplast could be crucial for preserving vacuolar integrity under stress conditions. Finally, we performed proximity-labelling proteomics of the TurboID-tagged ATG8A line upon monensin treatment. We found a significant overlap with the ES20-1 proxitome, further supporting our live cell imaging results (Fig. 6d).

**Fig. 7:**
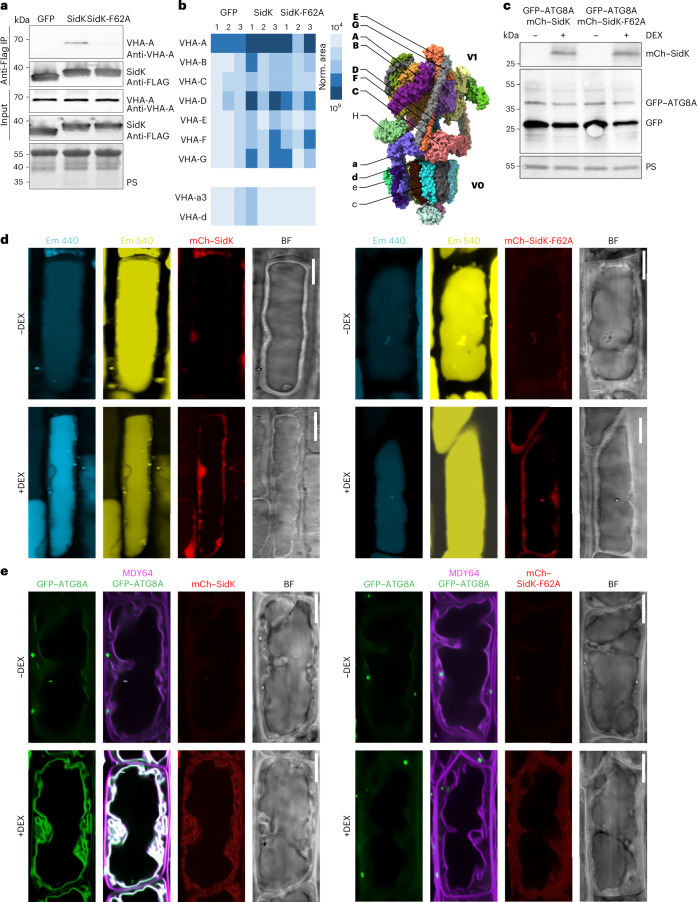
SidK expression induces tonoplast ATG8ylation in *A. thaliana.* **a**, Western blot analysis from immunoprecipitation (IP) experiments using Flag–GFP, Flag–SidK or Flag–SidK-F62A to pull down the VHA-A subunit of V-ATPase. The blots were probed with anti-Flag and anti-VHA-A antibodies to detect the presence of VHA-A in the pull-down from each bait protein. **b**, IP followed by mass spectrometry (IP–MS) results, presenting the identification of V-ATPase subunits co-immunoprecipitated with Flag–SidK and Flag–SidK-F62A. A schematic representation of the V-ATPase complex is also shown, detailing all its subunits. The ones marked with bold letters were detected in the SidK IP–MS experiment. **c**, Inducible expression of SidK as a tool to probe V-ATPase function in *Arabidopsis* cells. Western blot analysis of GFP–ATG8A lines expressing either mCh–SidK or mCh–SidK-F62A under a DEX-inducible promoter is presented, showing protein levels with and without DEX induction. The blots were probed for anti-GFP and mCh to detect the fusion proteins. **d**, SidK expression changes vacuolar pH. Confocal micrographs of mCh–SidK or mCh–SidK-F62A expressing lines treated with LysoSensor Yellow/Blue DND-160 are shown, displaying blue, yellow and red emissions, alongside bright field images, with and without DEX induction. Representative images from ten seedlings were analysed under each treatment. Scale bars, 10 µm. **e**, SidK expression induces tonoplast ATG8ylation. Confocal micrographs of GFP–ATG8A co-expressing mCh–SidK or mCh–SidK-F62A, treated with the tonoplast marker MDY64 displayed in magenta, are shown. Images show the GFP channel, a merge of the magenta and green channels, red emissions and bright field, with and without DEX induction. Representative images from ten seedlings were analysed under each treatment. Scale bars, 10 µm. Source data

We next sought to functionally probe the role of V-ATPase in tonoplast ATG8ylation. V-ATPase function is essential in plants, and the available genetic mutants have pleiotropic phenotypes^44^. To functionally probe the role of V-ATPase in tonoplast ATG8ylation, we decided to develop the *Legionella* effector protein SidK as a tool to study V-ATPase function in a spatiotemporally controlled manner. SidK binds the budding yeast VHA-A subunit and triggers the constitutive assembly of the V_1_ and V_0_ subcomplexes, leading to the inhibition of V-ATPase activity^45^. First, we tested whether SidK interacts with the plant V-ATPase. We expressed Flag-tagged SidK and its non-V-ATPase-binding mutant, SidK-F62A, in *Escherichia coli* and performed immunoprecipitation with *A. thaliana* lysates. Western blot analysis of the pull-down revealed that SidK but not SidK-F62A interacts with the VHA-A subunit of V-ATPase (Fig. 7a). Further mass spectrometry analysis of the immunoprecipitants also confirmed the presence of other V-ATPase subunits, demonstrating that SidK is a potential tool to study V-ATPase function in plants (Fig. 7b and Supplementary Table 2).

We then expressed SidK and SidK-F62A in *Arabidopsis* in a dexamethasone (DEX)-inducible manner (Fig. 7c). Lysosensor staining showed that SidK expression increased vacuolar pH, while SidK-F62A expression did not have a measurable effect (Fig. 7d). Crucially, SidK expression mimicked ES20-1 and monensin treatments and led to the colocalization of GFP–ATG8A with the tonoplast stain MDY-64 (Fig. 7e). This effect was dependent on binding to V-ATPase, since the expression of SidK-F62A did not change the localization of GFP–ATG8A. Altogether, these results demonstrate that SidK is a valuable tool for dissecting V-ATPase function in plants, and V-ATPase has a key role in tonoplast ATG8ylation.

## Discussion

**Fig. 8:**
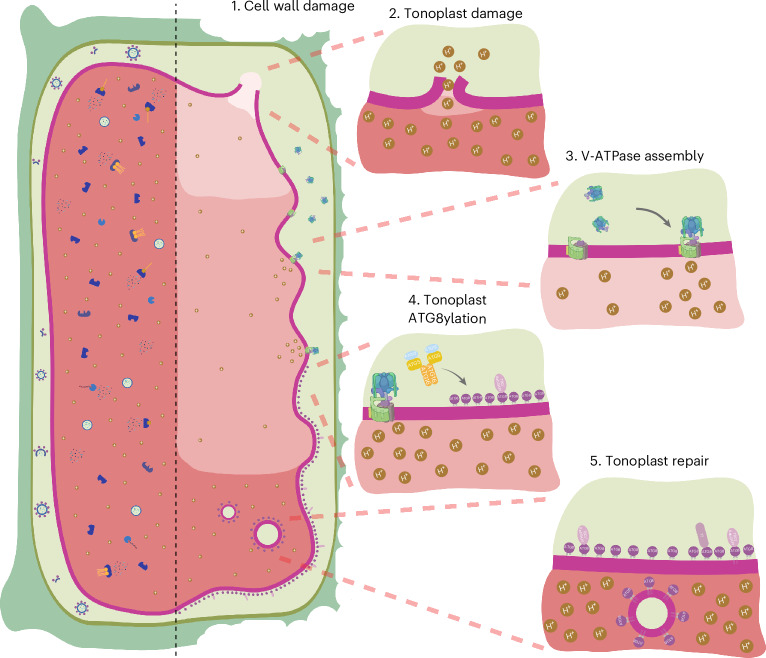
Model of the plant response to vacuolar damage upon cell wall damage through ATG8ylation of the tonoplast. Tonoplast ATG8ylation is a vacuolar quality control mechanism. After cell wall damage (1), the vacuole gets damaged (2). This induces the assembly of the V-ATPase (3) and subsequent tonoplast ATG8ylation (4). This facilitates tonoplast repair (5). Figure created with BioRender.com.

Here we define a conserved VQC mechanism that protects plant cells against the deleterious intracellular consequences of cell wall damage (Fig. 8). In contrast to cell wall stiffening (which induces autophagy), the inhibition of cellulose biosynthesis or enzymatic degradation of the cell wall (which mimics fungal infection) triggers turgor-pressure-dependent ATG8ylation of the tonoplast (Fig. 1). This is a clear example of CASM, which has been demonstrated for various cellular compartments in mammalian cells as a stress response mediating membrane remodelling and in plant cells mediating Golgi recovery after heat stress^20,46,47^. Notably, tonoplast ATG8ylation is different from the vacuolar ATG8 localization observed in yeast during stress^48,49^, since ATG8 is covalently conjugated to the tonoplast and depends on the ATG8 conjugation pathway (Fig. 2). Interestingly, cell wall damage does not trigger the relocation of the key ESCRT proteins VPS23 or ALIX to the tonoplast (Extended Data Fig. 9). Considering the well-established role of ESCRT in membrane repair^50^, further systematic studies are necessary to test whether other ESCRT proteins have a role in cell-wall-damage-induced VQC or whether ESCRT-dependent branches of VQC are activated under other stress conditions.

Genetic analyses of tonoplast ATG8ylation revealed that ATG11, an essential protein for selective autophagy, is not required, but ATG8 conjugation machinery, particularly the WD40 domain of ATG16, is essential (Fig. 2). Ultrastructural analysis of the *ΔCASM* line, which is defective only in tonoplast ATG8ylation, but not in autophagy, showed that cell wall damage triggers alterations of the vacuolar morphology and cell death (Fig. 3). A previous study using yeast ATG8 has shown that ATG8 lipidation and membrane attachment could cause tubulation and budding of liposomes^43^. ATG8ylation of the tonoplast could isolate damaged compartments of the vacuole from the rest to maintain vacuolar integrity. Consistently, we saw fragmented vacuoles in the *ΔCASM* line (Fig. 3). Further characterization of the *ΔCASM* line, together with various proteins that we identified in our proximity-labelling experiments, will be crucial to uncovering other molecular players involved in VQC (Fig. 4b–d).

The main trigger for tonoplast ATG8ylation is probably an increase in vacuolar pH linked to V-ATPase assembly. All three inducers of tonoplast ATG8ylation—cell wall damage, ionophore treatment and SidK expression—increase vacuolar pH (Figs. 4–7). Similar to mammalian cells, the V-ATPase–ATG16 axis executes tonoplast ATG8ylation upon pH changes^20,51,52^. However, how the plant cell senses the changes in vacuolar pH needs further investigation.

Moving forward, a key gap that needs to be filled is the molecular connection between CWI and VQC. The mechanical stability of a plant cell is safeguarded by intricate mechanostasis (mechanical homeostasis) pathways that balance two opposing mechanical forces: the outward turgor pressure of the cell and the inward forces of the elastic cell wall^13^. Defects in one of the two balancing factors will destabilize the balance and threaten plant fitness. So far, the effect of cell wall damage has been studied in relation to the repair of the cell wall. How the cell adjusts the turgor pressure upon cell wall damage has remained elusive. Our findings suggest a vacuolar mechanostasis pathway that promptly adjusts the turgor pressure upon cell wall damage to prevent cellular rupture (Fig. 3). Consistently, the key mechanosensory channel protein PIEZO localizes at the tonoplast rather than the plasma membrane in plants^53^. Although further studies are necessary to test whether PIEZO is sensitive to cell wall damage or whether tonoplast ATG8ylation is regulated by PIEZO, these findings place vacuoles as central hubs for mechanostasis in plants.

Our findings suggest that tonoplast ATG8ylation is independent of FERONIA. A systematic analysis of the CWI sensors (particularly the cellulose integrity sensor THESEUS), sensors that respond to pH changes and mechanosensitive ion channels will be crucial to uncovering the links between CWI and ATG8ylation^54^. Further elucidation of VQC pathways will reveal the connections between VQC, the cell wall and other cellular compartments that play a role in mechanostasis and reveal how they trigger VQC mechanisms to maintain cellular homeostasis.

## Methods

### Plant material and cloning procedure

All *A. thaliana* lines used in this study originate from the Col0 ecotype background and are listed in Supplementary Table 3. All transgenic lines were generated by floral dipping^55^, and plasmid constructs were cloned with the GreenGate cloning method^56^. The coding sequences of genes of interest were ordered from Twist Biosciences. A list of the plasmids produced is provided in Supplementary Table 4. *pATG8E*::*mCherry–TurboID*, *pATG8E*::*mCherry–TurboID–ATG8E* and *pATG8E*::*mCherry–TurboID–ATG8E*^*ADS*^ lines were subjected to reverse transcription PCR (RT–PCR), and homozygous plants with similar expression levels of TurboID were selected. For DEX-induced expression of SidK in *Arabidopsis* roots, the full-length sequence of SidK or SidK-F62A was synthesized (Twist) and subsequently cloned by GreenGate cloning with a C-terminal mCherry tag under the control of 3xOPp into Module N. *GR-LhG4* expression was driven by the UBI10 promotor and cloned in Module M. Modules M and N were combined in the destination vector pGGZ003 and transformed into *Arabidopsis* following *Agrobacterium*-mediated standard protocols. APEX2–ATG8A was cloned via GreenGate cloning into pGGSun with a UBI10 promoter. Homozygous plants with similar expression levels of APEX2 were selected. The knockout mutant *atg16* was created via CRISPR–Cas9-mediated mutation^57^. The plasmid pCBCDT1T2 was used as the scaffold template and pHEE401E as the destination vector. The guides, designed via CRISPR-P v.2.0 (http://crispr.hzau.edu.cn/cgi-bin/CRISPR2/CRISPR), are GATCGGGAAACCATTGGCAT and GGTACAGGAGGAGAAAGCTA, which target the beginning of the second exon and the end of the fifth and last exon, respectively. A mutant lacking the whole region was obtained, Δ107–2183. After homozygous lines were obtained in T_1_, Cas9 was crossed out with a Col0 line, and a new homozygous T_2_ Cas9-free plant was selected. *ATG16* was cloned via GreenGate cloning into pGGZ003 with a UBI10 promoter. *ATG16* complementation constructs were cloned via PCR from the previous vector. Each PCR (*ATG16*^1–195^, *ATG16*^1–253^, *ATG16*^1–295^ and *ATG16*^1–379^) became a module, which was cloned via GreenGate cloning into pGGSun with a UBI10 promoter. *ATG16* and the four other delta C deletions were transformed into the *atg16* mutant background, and homozygous plants were selected in T_2_ generation.

Male *M. polymorpha* Takaragaike-1 plants were maintained asexually and cultured through gemma on half-strength Gamborg’s B5 medium supplemented with 0.5 g l^−1^ MES, 1% sucrose and 1% agar under 50 µM m^−^^2^ s^−1^ continuous white light at 21 °C. The plant lines used are listed in Supplementary Table 3.

### Plant growth and plant treatments

For standard plant growth, seeds were sown on water-saturated soil and kept under a 16 h light/8 h dark photoperiod with 165 µmol m^−2^ s^−1^ light intensity. For in vitro seedling growth, *Arabidopsis* seeds were surface-sterilized in 70% ethanol and 0.05% SDS for 15 min, rinsed in ethanol absolute and dried on sterile paper. Seeds were plated in ½ Murashige and Skoog (MS) salts (Duchefa)/1% agar/1% sucrose plates and stratified for 48 h in the dark at 4 °C. The plates were then kept under LEDs with 50 µM m^−^^2^ s^−1^ and a 16 h light/8 h dark photoperiod for the indicated amount of time.

For drug treatments, all drugs used were dissolved in DMSO (unless stated otherwise) and added to the desired concentration: 9 μM Torin1 (CAS 1222998-36-8, Santa Cruz), 50 µM EGCG (E4143, Sigma-Aldrich), 100 µM ES20 and ES20-1 (ref. ^17^), 3 nM isoxaben on MS agar plates (82558-50-7, Sigma-Aldrich), 1% Driselase in PBS (85186-71-6, Sigma-Aldrich), 50 mM sorbitol, 200 µM monensin (22373-78-0, Sigma-Aldrich) and DEX on MS agar plates (50-02-2, Sigma-Aldrich). An equal amount of pure DMSO, or the respective solvent, was added to the control samples. For pH treatments, different buffers were applied. For low pHs (5 and 6), the main component was 50 mM MES; for high pHs (7, 7.5 and 8), it was 50 mM HEPES. All buffers were enriched with 50 mM ammonium acetate and BTP or HCl to reach the desired pH.

### Carbon starvation assay

A total of 30–40 *A. thaliana* seeds were surface-sterilized with ethanol, vernalized for two days at 4 °C in the dark and grown in ½ MS media (MS salt + Gamborg B5 vitamin mixture (Duchefa) supplemented with 0.5 g l^−1^ MES and 1% sucrose, pH 5.7) for nine days at 21 °C under LEDs with 85 µM m^−2^ s^−1^ with a 14 h light/10 h dark photoperiod. Nine-day-old seedlings were rinsed twice with new carbon-depleted ½ MS media (MS salt + Gamborg B5 vitamin mixture (Duchefa) supplemented with 0.5 g l^−1^ MES, pH 5.7) and incubated for four days with the carbon-depleted media in the dark. Control seedlings were rinsed twice with ½ MS media and incubated for four days under LEDs with 85 µM m^−2^ s^−1^ with a 14 h light/10 h dark photoperiod. Pictures were taken on the fourth day of treatment with a Canon EOS 80D.

### Preparation of *M. polymorpha* samples for confocal microscopy

The *M. polymorpha* asexual gemmae were incubated in liquid ½ Gamborg B5 media for two days before imaging. Two-day-old *M. polymorpha* thalli were placed on a microscope slide with deionized water and covered with a coverslip. The meristem region was used for image acquisition.

### Confocal microscopy

All images were acquired via an inverted point laser scanning confocal microscope (LSM800, Carl Zeiss) equipped with high-sensitive GaAsP (Gallium Arsenide, like LSM780 and 880) detectors, a transmitted light detector, a ×20/0.8 plan-apochromat DIC, a ×63/1.2 plan-apochromat (water immersion) and ZEN software (blue edition, Carl Zeiss). The ×63 objective was used for all images except Fig. 3f, for which the ×20 objective was used instead. GFP and YFP fluorescence were excited at 488 nm and detected between 488 and 545 nm. MDY64 fluorescence was excited at 405 nm and detected between 465 and 550 nm. RFP, mCherry and PI fluorescence were excited at 561 nm and detected between 570 and 617 nm. Yellow/Blue DND-160 was excited at 405 nm and detected between 400 and 500 nm for blue detection and 500 and 600 nm for yellow detection. For *Z*-stack imaging, the interval between the layers was set at 1 μm. For each experiment, all replicate images were acquired using identical confocal microscopic parameters. The confocal images were processed with Fiji (v.1.54, Fiji).

Different markers were applied for 10 min before visualizing the samples when indicated in the figures: 50 μg ml^−1^ PI in PBS (25535-16-4, Sigma-Aldrich), 1 μM MDY-64 (Y7536, Invitrogen) and 20 mM Lysosensor Yellow/Blue DND-160 (L22460, Invitrogen). Mechanoprobes were also applied before measurements: 10 μM CarboTag-BDP for 30 min, 10 μM Sulfo-BDP for 30 min and 10 μM PEG-BDP for 90 min.

### Image processing and quantification

Pearson’s and Spearman’s colocalization analyses were performed with Fiji (v.1.54, Fiji). Puncta quantification was performed with Fiji (v.1.54, Fiji). *Z*-stack images (at least five layers) were background-subtracted with 25 pixels of rolling ball radius. Each *Z*-stack image was subsequently thresholded using the MaxEntropy method and was converted to an eight-bit greyscale image. Threshold values were adjusted according to the puncta signals in the original confocal images. The number of puncta in the thresholded images was counted via the Analyze Particles function in Fiji. For all puncta quantification, puncta with sizes between 0.10 and 4.00 μm^2^ were counted.

The vacuolar morphology index was quantified in six‐day‐old seedlings. Confocal images were analysed using Fiji (v.1.54). To calculate the vacuolar morphology index, the longest and widest distance of the biggest luminal structure was measured and multiplied.

### APEX2 labelling and electron microscopy sample preparation

After the respective plant treatments (Torin or ES20-1), the roots of seedlings were dissected (on ice) in 2.5% glutaraldehyde in 0.1 M cacodylate buffer (pH 7.4) and incubated in this fixative solution for 1 h under vacuum. After the samples were washed thoroughly with 0.1 M cacodylate buffer (pH 7.4; four or five times), the specimens were incubated in fresh prepared DAB solution with H_2_O_2_ (DAB 0.5 mg ml^−1^, H_2_O_2_ 10 mM) in 0.1 M cacodylate buffer^18^ for 50 min (on ice covered with tinfoil). Some samples were left without DAB treatment in this step to use as a negative control. Then, the DAB solution was gently removed, and the specimens were washed with 0.1 M cacodylate buffer three times for 1 min. After that, the specimens were incubated in fresh prepared 1% (w/v) OsO_4_ in 0.1 M cacodylate buffer for 40 min at room temperature. Excess OsO_4_ was washed with deionized water (four or five times, 15 min for each step); then, the deionized water was gently removed, and the specimens were submerged in 2% (w/v) uranyl acetate solution for 50 min covered with tinfoil. The excess uranyl acetate was then washed with deionized water (four or five times, 15 min for each step), and the specimens were dehydrated with a graduated acetone series in deionized water (from 10% to 100% acetone; 30 min for each step). The samples were then embedded in Embed-812 resin (cat. no. 14120, Electron Microscopy Sciences) and polymerized in a 60 °C oven for 24 h. Ultrathin sections (70 nm thick) were prepared from the sample blocks. The sections were examined with a transmission electron microscope (Morgagni 268) operated at 80 kV.

### Observation of the tonoplast via transmission electron microscopy and electron tomography

After the respective plant treatments, the roots of seedlings were dissected (on ice) in 2.5% glutaraldehyde in 0.1 M cacodylate buffer (pH 7.4) and incubated in this fixative solution overnight at 4 °C. After the samples were washed thoroughly with 0.1 M cacodylate buffer (pH 7.4; four or five times), the specimens were postfixed by incubation in fresh prepared 1% (w/v) OsO_4_ in 0.1 M cacodylate buffer for 1 h at room temperature. Excess OsO_4_ was washed with deionized water (four or five times, 15 min for each step), and the samples were dehydrated with a graduated acetone series in deionized water (from 10% to 100% acetone; 30 min for each step). The samples were then embedded in Embed-812 resin and polymerized in a 60 °C oven for 24 h. Thin sections (90 nm thick) were prepared from the sample blocks. The sections were post-stained and examined with a transmission electron microscope (Morgagni 268) operated at 80 kV.

A series of semi-thick sections (250 nm) were collected on a copper slot grid (cat. no. GS2010-Cu, Electron Microscopy Sciences). After post-staining and gold particle coating, tilt series were collected with a 200 kV Tecnai G2 20 electron microscope (+50° to −50° at an interval of 1° around two orthogonal axes). Tomogram calculation and 3D model rendering were performed with the IMOD software package as described previously^58,59^.

### FLIM

The FLIM imaging experiments were performed on a Picoquant Fluorescent Lifetime Imaging Microscope equipped with an Olympus iX71 inverted microscope frame, a PL ×20 Plan Achromat Objective, numerical aperture (NA) = 0.4, NA = 1.2 (water immersion), a Hybrid Photomultiplier Detection Assembly with <50 ps time resolution and SymPhoTime 64 (Picoquant). The samples were excited with a 488 nm pulsed laser source (pulse duration, <1 ps). Acquisition time was fixed at 120 s for each 256 × 256 pixel image. The FLIM images were processed using SymPhoTime 64 software to fit the fluorescence decay curves in each pixel with a two-component exponential decay. The images are reported in a false-colour scale that represents the mean fluorescence lifetime for each pixel, expressed in nanoseconds. Three technical replicates were measured by session, and their average is represented in the final graph. Three biological replicates were measured on different days, and their average mean value is provided as the final value.

### Protein extraction and western blotting

A total of 20–40 *A. thaliana* seeds were surface-sterilized with ethanol, vernalized for two days at 4 °C in the dark and grown in ½ MS media (MS salt + Gamborg B5 vitamin mixture (Duchefa) supplemented with 0.5 g l^−1^ MES and 1% sucrose, pH 5.7) for seven days at 21 °C under LEDs with 85 µM m^−2^ s^−1^ with a 14 h light/10 h dark photoperiod. For drug treatments, monensin (CAS 22373-78-0, Sigma-Aldrich) was dissolved in pure ethanol, and Torin1 (CAS 1222998-36-8, Santa Cruz) and ES20-1 (refs. ^60^) were dissolved in DMSO; they were then added to the desired concentrations: 200 mM monensin, 3 μM Torin1 and 100 μM ES20-1. Equal amounts of pure ethanol or DMSO were added to mock samples. Seedlings were harvested in safe-lock Eppendorf tubes containing 2 mm Ø glass beads, flash-frozen in liquid nitrogen and ground using a Silamat S7 (Ivoclar vivadent). Total proteins were extracted in 2× Laemmli buffer by shaking again in the Silamat S7 for 20 s. The samples were boiled for 10 min at 70 °C and shaken, then centrifuged for 5 min at maximum speed. Total proteins were quantified with the amido black method. Next, 10 μl of supernatant was mixed with 190 μl of deionized water and added to 1 ml of amido black buffer (10% acetic acid, 90% methanol, 0.05% (w/v) amido black (Naphtol Blue Black, Sigma N3393)), mixed and centrifuged for 10 min at maximum speed. The pellets were then washed with 1 ml of wash buffer (10% acetic acid and 90% ethanol), mixed and centrifuged for 10 min at maximum speed and resuspended in 0.2 N NaOH. OD_630 nm_ was measured, with NaOH solution as the blank, and protein concentration was calculated using the OD = *a*[C] + *b* determined curve. Then, 2.5–40 μg of total protein extracts were separated on SDS–PAGE gels and blotted onto PVDF Immobilon-P membrane (Millipore). GFP was detected using the anti-GFP antibody (mouse monoclonal, 11814460001, Roche) diluted 1:5,000 (v/v). NBR1 was detected using the anti-NBR1 antibody (rabbit polyclonal, AS14 2805, Agrisera) diluted 1:5,000 (v/v). Subunit A of V-ATPase was detected using the anti-V-ATPase-A antibody (rabbit polyclonal) diluted 1:5,000 (v/v). Flag was detected using anti-Flag M2 antibody (mouse monoclonal, F3165, Sigma) diluted to 10 μg ml^−1^. mCherry was detected using anti-RFP (mouse monoclonal, AB_2631395, Chromotek) diluted 1:2,000 (v/v). Mouse monoclonal antibodies were detected with goat anti-mouse IgG HRP-linked antibody (61-6520, Invitrogen) diluted 1:5,000 (v/v). Rabbit polyclonal antibody was detected with goat anti-rabbit IgG HRP-linked antibody (65-6120, Invitrogen) diluted 1:5,000. Hybridized membranes were reacted with SuperSignal West Pico PLUS Chemiluminescent Substrate (Thermo Fisher Scientific) and imaged using an iBright CL1500 Imaging System (Invitrogen).

### Pull-down of plant V-ATPase from plant extracts

To pull down native V-ATPase from plant extracts, *Arabidopsis* leaf tissue was collected and ground to fine powder in liquid nitrogen using a pestle and mortar. The leaf powder was mixed with two times volume/weight ice-cold extraction buffer (10% glycerol, 25 mM Tris pH 7.5, 1 mM EDTA, 150 mM NaCl, 2% w/v PVPP, 10 mM DTT, 0.2% IGEPAL (Merck)) supplemented with EDTA-free protease inhibitor tablets (Roche) and centrifuged at 5,000 *g* at 4 °C for 20–30 min. The supernatant was passed through a 0.45 μm Minisart syringe filter.

For immunoprecipitation, 6xHis–GFP–3xFlag, 6xHis–SidK–3xFlag or 6xHis–SidKF62A–3xFlag protein was added to 1 ml of filtered extract to a final concentration of ~2.5 μM and incubated in a rotatory mixer at 4 °C with 25 μl of Anti-Flag M2 Magnetic Beads (Merck). Then, 30 μl of the mixture was taken as input for western blot analysis before adding magnetic beads. After 2.5 h the beads were pelleted using a magnetic rack, and the supernatant was removed. The pellets were washed by resuspension in 1 ml of IP buffer (10% glycerol, 25 mM Tris pH 7.5, 1 mM EDTA, 150 mM NaCl, 0.2% IGEPAL (Merck)) and pelleted again in the magnetic rack. The washing steps were repeated three times with IP buffer and three times with 50 mM Tris pH 7.5, 150 mM NaCl.

Finally, the beads were pelleted by centrifugation and incubated for 10 min at 70 °C with SDS loading buffer. The beads were then pelleted again, and the supernatant was loaded on SDS–PAGE gels prior to western blotting. Membranes were probed with anti-Flag M2 antibody (Merck) to detect SidK and anti-A (AS09 467, Agrisera) to detect subunit A of V-ATPase.

### In vivo co-immunoprecipitation

For co-immunoprecipitation, 14 mg of seeds per sample were grown in ½ MS media for seven days. Proteins were extracted by adding three equivalent volumes of extraction buffer (10% glycerol, 10 mM DTT, 10 mM Tris pH 7.4, 50 mM KCl, 5 mM MgCl2, 5 mM ATP, 0.5% dodecyl beta-d-maltoside, 20 μg ml^−1^ Pepstatin, 1 tablet per 50 ml cOmplete EDTA-free Protease Inhibitor Cocktail (Roche)). Lysates were cleared by centrifugation at 1,500 *g* at 5 °C for 15 min three times. After the first centrifugation, the supernatant was filtered with Miracloth (Sigma-Aldrich). The supernatant was incubated with 30 μl of GFP-Trap Agarose beads (Chromotek) for 1.5 h. The beads were washed three times with wash buffer (10% glycerol, 10 mM DTT, Tris pH 7.4, 50 mM KCl, 2 mM MgCl2, 1 mM ATP, 0.1% dodecyl beta-d-maltoside, 20 μg ml^−1^ Pepstatin, 1 tablet per 50 ml cOmplete EDTA-free Protease Inhibitor Cocktail (Roche)) before and after incubation with lysate. The beads were eluted in 100 μl of 2× Laemmli buffer, boiled for 5 min at 95 °C and subjected to western blot with indicated antibodies.

### ES20-1 synthesis

*o*-Methyl benzoyl hydrazine (3.00 g, 20 mmol, 1.00 equiv.) was dissolved in 80 ml of absolute ethanol under stirring, and benzoyl isothiocyanate (3.26 g, 20 mmol, 1.00 equivalent) was added. After some minutes of stirring, a precipitate formed. The mixture was heated to reflux for 15 min. The solution was then allowed to cool to room temperature, at which point colourless crystals started to form. To complete crystallization, the flask containing the crystals and the mother liquor was cooled in an ice bath. The product was filtered, washed with cold ethanol and dried in vacuo. The product (4.90 g, 15.6 mmol, 78% yield) was obtained as an off-white crystalline solid.

^1^H NMR (700 MHz, CDCl3): *δ* = 13.45 (*s*, 1H), 9.48 (*s*, 1H), 9.05 (*s*, 1H,), 7.92–7.88 (*m*, 2H), 7.69–7.62 (*m*, 1H), 7.59 (*d*, 1H, *J* = 7.55 Hz), 7.56–7.51 (*m*, 2H), 7.45–7.39 (*m*, 1H), 7.31–7.27 (*m*, 2H), 2.56 (*s*, 3H) ppm.

^13^C NMR (176 MHz, CDCl3): *δ* = 171.35, 166.56, 164.46, 137.87, 133.83, 131.60, 131.46, 131.40, 131.06, 129.21, 127.61, 127.53, 126.03, 20.19 ppm.

High-resolution mass spectrometry (electrospray ionization): *m*/*z* calculated. For [C16H15N3O2S, M+Na]+: 336.0777; found: 336.0767.

### Phylogenetic analysis of TBC/RabGAP1

To build a phylogeny of TBC/RabGAP1, we first used BLASTP from the BLAST+ suite^61^ to search for sequences closely related to AT5G52580.1 in the *Arabidopsis* Information Resource database, the Solanaceae Genomics Network (https://solgenomics.net; genomes: Niben101 and Capang) and Phytozome (https://phytozome-next.jgi.doe.gov; genomes: A.thaliana_Araport11, A.lyratav2.1, C.rubellav1.1, E.salsugineumv1.0, T.cacaov2.1, P.vulgarisv2.1, G.maxWm82.a4.v1, M.truncatulaMt4.0v1, L.japonicusLj1.0v1, S.lycopersicumITAG5.0, S.tuberosumv6.1, A.comosusv3, A.trichopodav1.0, P.virgatumv5.1, S.bicolorv3.1.1, Z.maysRefGen_V4, O.sativav7.0, H.vulgare_MorexV3, T.aestivumv2.2, B.distachyonv3.1, M.polymorphav3.1, S.moellendorffiiv1.0, C.reinhardtii_CC-4532v6.1 and P.patensv3.3). In total, we collected 49 non-redundant sequences from 26 species (Supplementary Dataset 1). Amino-acid-based alignment was generated using MUSCLE^62^ and was subsequently trimmed from poorly aligned positions using Gblocks^63^ with less stringent parameters as implemented in http://phylogeny.lirmm.fr/phylo_cgi/. The resulting blocks were used to compute a maximum likelihood phylogenetic tree using IQ-Tree v.2 (ref. ^64^). The best-scoring tree was visualized using the iToL tool v.6.9 (refs. ^35,36,65^) and is publicly available at https://itol.embl.de/export/1931711883132731712669776.

### Reporting summary

Further information on research design is available in the Nature Portfolio Reporting Summary linked to this article.

## Supplementary information

Supplementary Information Supplementary Methods.

Reporting Summary

Supplementary Video 1 Electron tomography analysis of a *ΔCASM* root cell treated with ES20-1 (eight hours, 100 µM), providing a detailed three-dimensional visualization of vacuolar morphology and the surrounding cellular environment. Scale bar, 5 µm.

Supplementary Tables Supplementary Tables 1–4.

Supplementary Dataset 1 Sequences closely related to AT5G52580.1 from the *Arabidopsis* Information Resource database, Solanaceae Genomics Network and Phytozome. In total, 49 non-redundant sequences from 26 species are included.

## Source data

Source Data Fig. 2 Unprocessed western blots.

Source Data Fig. 5 Unprocessed western blots.

Source Data Fig. 7 Unprocessed western blots.

Source Data Extended Data Fig. 4 Unprocessed western blots.

## Data Availability

Source data are provided with this paper. These data are also available via Zenodo at 10.5281/zenodo.10993280 (ref. ^66^). The *Arabidopsis* reference genome was obtained from TAIR10 (https://www.arabidopsis.org). The other reference genomes were obtained from Phytozome 13 (https://phytozome-next.jgi.doe.gov/) and the Solanaceae Genomics Network (https://solgenomics.sgn.cornell.edu/).

## References

[1] Braidwood, L., Breuer, C. & Sugimoto, K. My body is a cage: mechanisms and modulation of plant cell growth. *N. Phytol.* **201**, 388–402 (2014). 10.1111/nph.12473 24033322

[2] Vaahtera, L., Schulz, J. & Hamann, T. Cell wall integrity maintenance during plant development and interaction with the environment. *Nat. Plants* **5**, 924–932 (2019). 31506641 10.1038/s41477-019-0502-0

[3] Cosgrove, D. J. Structure and growth of plant cell walls. *Nat. Rev. Mol. Cell Biol.* **25**, 340–358 (2023). 38102449 10.1038/s41580-023-00691-y

[4] Wolf, S. Cell wall signaling in plant development and defense. *Annu. Rev Plant Biol.* **73**, 323–353 (2022). 35167757 10.1146/annurev-arplant-102820-095312

[5] Bacete, L. & Hamann, T. The role of mechanoperception in plant cell wall integrity maintenance. *Plants (Basel)* **9**, 574 (2020). 32369932 10.3390/plants9050574 PMC7285163

[6] Baez, L. A., Ticha, T. & Hamann, T. Cell wall integrity regulation across plant species. *Plant Mol. Biol.* **109**, 483–504 (2022). 35674976 10.1007/s11103-022-01284-7 PMC9213367

[7] Kruger, F. & Schumacher, K. Pumping up the volume—vacuole biogenesis in *Arabidopsis thaliana*. *Semin. Cell Dev. Biol.* **80**, 106–112 (2018). 28694113 10.1016/j.semcdb.2017.07.008

[8] Marty, F. Plant vacuoles. *Plant Cell* **11**, 587–600 (1999). 10213780 10.1105/tpc.11.4.587 PMC144210

[9] Dunser, K. et al. Extracellular matrix sensing by FERONIA and Leucine-Rich Repeat Extensins controls vacuolar expansion during cellular elongation in *Arabidopsis thaliana*. *EMBO J.* **38**, e100353 (2019). 30850388 10.15252/embj.2018100353 PMC6443208

[10] Scheuring, D. et al. Actin-dependent vacuolar occupancy of the cell determines auxin-induced growth repression. *Proc. Natl Acad. Sci. USA* **113**, 452–457 (2016). 26715743 10.1073/pnas.1517445113 PMC4720293

[11] Lofke, C., Dunser, K., Scheuring, D. & Kleine-Vehn, J. Auxin regulates SNARE-dependent vacuolar morphology restricting cell size. *eLife* **4**, e05868 (2015). 25742605 10.7554/eLife.05868 PMC4384535

[12] Engelsdorf, T. et al. The plant cell wall integrity maintenance and immune signaling systems cooperate to control stress responses in *Arabidopsis thaliana*. *Sci. Signal.* **11**, eaao3070 (2018). 29945884 10.1126/scisignal.aao3070

[13] Codjoe, J. M., Miller, K. & Haswell, E. S. Plant cell mechanobiology: greater than the sum of its parts. *Plant Cell* **34**, 129–145 (2022). 34524447 10.1093/plcell/koab230 PMC8773992

[14] Chang, C., Jensen, L. E. & Hurley, J. H. Autophagosome biogenesis comes out of the black box. *Nat. Cell Biol.* **23**, 450–456 (2021). 33903736 10.1038/s41556-021-00669-y PMC8122082

[15] Mizushima, N. The ATG conjugation systems in autophagy. *Curr. Opin. Cell Biol.* **63**, 1–10 (2020). 31901645 10.1016/j.ceb.2019.12.001

[16] Klionsky, D. J. et al. Guidelines for the use and interpretation of assays for monitoring autophagy (4th edition)(1). *Autophagy* **17**, 1–382 (2021). 33634751 10.1080/15548627.2020.1797280 PMC7996087

[17] Huang, L., Li, X. & Zhang, C. Endosidin20-1 is more potent than endosidin20 in inhibiting plant cellulose biosynthesis and molecular docking analysis of cellulose biosynthesis inhibitors on modeled cellulose synthase structure. *Plant J.* **106**, 1605–1624 (2021). 33793980 10.1111/tpj.15258

[18] Martell, J. D., Deerinck, T. J., Lam, S. S., Ellisman, M. H. & Ting, A. Y. Electron microscopy using the genetically encoded APEX2 tag in cultured mammalian cells. *Nat. Protoc.* **12**, 1792–1816 (2017). 28796234 10.1038/nprot.2017.065 PMC5851282

[19] Rogov, V. V. et al. Atg8 family proteins, LIR/AIM motifs and other interaction modes. *Autophagy Rep.* **2**, 2188523 (2023). 10.1080/27694127.2023.2188523 PMC7615515 38214012

[20] Durgan, J. & Florey, O. Many roads lead to CASM: diverse stimuli of noncanonical autophagy share a unifying molecular mechanism. *Sci. Adv.* **8**, eabo1274 (2022). 36288315 10.1126/sciadv.abo1274 PMC9604613

[21] Zou, Y. et al. ATG8 delipidation is not universally critical for autophagy in plants. *Nat. Commun.* **16**, 403 (2025). 39757240 10.1038/s41467-024-55754-1 PMC11701075

[22] Fujita, N. et al. The Atg16L complex specifies the site of LC3 lipidation for membrane biogenesis in autophagy. *Mol. Biol. Cell* **19**, 2092–2100 (2008). 18321988 10.1091/mbc.E07-12-1257 PMC2366860

[23] Dooley, H. C. et al. WIPI2 links LC3 conjugation with PI3P, autophagosome formation, and pathogen clearance by recruiting Atg12-5-16L1. *Mol. Cell* **55**, 238–252 (2014). 24954904 10.1016/j.molcel.2014.05.021 PMC4104028

[24] Fletcher, K. et al. The WD40 domain of ATG16L1 is required for its non-canonical role in lipidation of LC3 at single membranes. *EMBO J.* **37**, e97840 (2018). 29317426 10.15252/embj.201797840 PMC5813257

[25] Rasmussen, N. L., Kournoutis, A., Lamark, T. & Johansen, T. NBR1: the archetypal selective autophagy receptor. *J. Cell Biol.* **221**, e202208092 (2022). 36255390 10.1083/jcb.202208092 PMC9582228

[26] Zhao, J. et al. Plant autophagosomes mature into amphisomes prior to their delivery to the central vacuole. *J. Cell Biol.* **221**, e202203139 (2022). 36260289 10.1083/jcb.202203139 PMC9584626

[27] Michels, L. et al. Complete microviscosity maps of living plant cells and tissues with a toolbox of targeting mechanoprobes. *Proc. Natl Acad. Sci. USA* **117**, 18110–18118 (2020). 32669427 10.1073/pnas.1921374117 PMC7395454

[28] Besten, M. et al. CarboTag: a modular approach for live and functional imaging of plant cell walls. Preprint at *bioRxiv*10.1101/2024.07.05.597952 (2024). 10.1038/s41592-025-02677-4 PMC12074989 40312511

[29] Gigli-Bisceglia, N. et al. Cell wall integrity modulates *Arabidopsis thaliana* cell cycle gene expression in a cytokinin- and nitrate reductase-dependent manner. *Development* **145**, dev166678 (2018). 30190280 10.1242/dev.166678

[30] Yang, H. & Tan, J. X. Lysosomal quality control: molecular mechanisms and therapeutic implications. *Trends Cell Biol.* **33**, 749–764 (2023). 36717330 10.1016/j.tcb.2023.01.001 PMC10374877

[31] Barisch, C., Holthuis, J. C. M. & Cosentino, K. Membrane damage and repair: a thin line between life and death. *Biol. Chem.* **404**, 467–490 (2023). 36810295 10.1515/hsz-2022-0321

[32] Skowyra, M. L., Schlesinger, P. H., Naismith, T. V. & Hanson, P. I. Triggered recruitment of ESCRT machinery promotes endolysosomal repair. *Science* **360**, eaar5078 (2018). 29622626 10.1126/science.aar5078 PMC6195421

[33] Radulovic, M. et al. ESCRT-mediated lysosome repair precedes lysophagy and promotes cell survival. *EMBO J.* **37**, e99753 (2018). 30314966 10.15252/embj.201899753 PMC6213280

[34] Kalinowska, K. et al. *Arabidopsis* ALIX is required for the endosomal localization of the deubiquitinating enzyme AMSH3. *Proc. Natl Acad. Sci. USA* **112**, E5543–E5551 (2015). 26324913 10.1073/pnas.1510516112 PMC4603487

[35] Gao, C. et al. A unique plant ESCRT component, FREE1, regulates multivesicular body protein sorting and plant growth. *Curr. Biol.* **24**, 2556–2563 (2014). 25438943 10.1016/j.cub.2014.09.014

[36] Nagel, M.-K. et al. *Arabidopsis* SH3P2 is an ubiquitin-binding protein that functions together with ESCRT-I and the deubiquitylating enzyme AMSH3. *Proc. Natl Acad. Sci. USA* **114**, E7197–E7204 (2017). 28784794 10.1073/pnas.1710866114 PMC5576839

[37] Bhattacharya, A. et al. A lysosome membrane regeneration pathway depends on TBC1D15 and autophagic lysosomal reformation proteins. *Nat. Cell Biol.* **25**, 685–698 (2023). 37024685 10.1038/s41556-023-01125-9

[38] Simon, M. L. et al. A multi-colour/multi-affinity marker set to visualize phosphoinositide dynamics in *Arabidopsis*. *Plant J.* **77**, 322–337 (2014). 24147788 10.1111/tpj.12358 PMC3981938

[39] Platre, M. P. et al. A combinatorial lipid code shapes the electrostatic landscape of plant endomembranes. *Dev. Cell* **45**, 465–480.e411 (2018). 29754803 10.1016/j.devcel.2018.04.011

[40] Cross, J. et al. Lysosome damage triggers direct ATG8 conjugation and ATG2 engagement via non-canonical autophagy. *J. Cell Biol.* **222**, e202303078 (2023). 37796195 10.1083/jcb.202303078 PMC10561555

[41] Otegui, M. S., Herder, R., Schulze, J., Jung, R. & Staehelin, L. A. The proteolytic processing of seed storage proteins in *Arabidopsis* embryo cells starts in the multivesicular bodies. *Plant Cell* **18**, 2567–2581 (2006). 17012602 10.1105/tpc.106.040931 PMC1626608

[42] Stenseth, K. & Thyberg, J. Monensin and chloroquine inhibit transfer to lysosomes of endocytosed macromolecules in cultured mouse peritoneal macrophages. *Eur. J. Cell Biol.* **49**, 326–333 (1989). 2776777

[43] Maruyama, T. et al. Membrane perturbation by lipidated Atg8 underlies autophagosome biogenesis. *Nat. Struct. Mol. Biol.* **28**, 583–593 (2021). 34239122 10.1038/s41594-021-00614-5

[44] Kriegel, A. et al. Job sharing in the endomembrane system: vacuolar acidification requires the combined activity of V-ATPase and V-PPase. *Plant Cell* **27**, 3383–3396 (2015). 26589552 10.1105/tpc.15.00733 PMC4707456

[45] Zhao, J. et al. Molecular basis for the binding and modulation of V-ATPase by a bacterial effector protein. *PLoS Pathog.* **13**, e1006394 (2017). 28570695 10.1371/journal.ppat.1006394 PMC5469503

[46] Deretic, V. & Lazarou, M. A guide to membrane atg8ylation and autophagy with reflections on immunity. *J. Cell Biol.* **221**, e202203083 (2022). 35699692 10.1083/jcb.202203083 PMC9202678

[47] Zhou, J. et al. A non-canonical role of ATG8 in Golgi recovery from heat stress in plants. *Nat. Plants* **9**, 749–765 (2023). 37081290 10.1038/s41477-023-01398-w

[48] Liu, X. M. et al. Lipidation-independent vacuolar functions of Atg8 rely on its noncanonical interaction with a vacuole membrane protein. *eLife* **7**, e41237 (2018). 30451685 10.7554/eLife.41237 PMC6279349

[49] Tamura, N., Oku, M. & Sakai, Y. Atg8 regulates vacuolar membrane dynamics in a lipidation-independent manner in *Pichia pastoris*. *J. Cell Sci.* **123**, 4107–4116 (2010). 21045113 10.1242/jcs.070045

[50] Zoncu, R. & Perera, R. M. Built to last: lysosome remodeling and repair in health and disease. *Trends Cell Biol.* **32**, 597–610 (2022). 35123838 10.1016/j.tcb.2021.12.009 PMC9189017

[51] Hooper, K. M. et al. V-ATPase is a universal regulator of LC3-associated phagocytosis and non-canonical autophagy. *J. Cell Biol.* **221**, e202105112 (2022). 35511089 10.1083/jcb.202105112 PMC9082624

[52] Xu, Y. et al. A bacterial effector reveals the V-ATPase-ATG16L1 axis that initiates xenophagy. *Cell* **178**, 552–566.e520 (2019). 31327526 10.1016/j.cell.2019.06.007

[53] Radin, I. et al. Plant PIEZO homologs modulate vacuole morphology during tip growth. *Science* **373**, 586–590 (2021). 34326243 10.1126/science.abe6310

[54] Bacete, L. et al. THESEUS1 modulates cell wall stiffness and abscisic acid production in *Arabidopsis thaliana*. *Proc. Natl Acad. Sci. USA* **119**, e2119258119 (2022). 34949719 10.1073/pnas.2119258119 PMC8740707

[55] Clough, S. J. & Bent, A. F. Floral dip: a simplified method for *Agrobacterium*-mediated transformation of *Arabidopsis thaliana*. *Plant J.* **16**, 735–743 (1998). 10069079 10.1046/j.1365-313x.1998.00343.x

[56] Lampropoulos, A. et al. GreenGate—a novel, versatile, and efficient cloning system for plant transgenesis. *PLoS ONE* **8**, e83043 (2013). 24376629 10.1371/journal.pone.0083043 PMC3869738

[57] Xing, H.-L. et al. A CRISPR/Cas9 toolkit for multiplex genome editing in plants. *BMC Plant Biol.* **14**, 327 (2014). 25432517 10.1186/s12870-014-0327-y PMC4262988

[58] Mai, K. K. K. & Kang, B. H. Semiautomatic segmentation of plant Golgi stacks in electron tomograms using 3dmod. *Methods Mol. Biol.* **1662**, 97–104 (2017). 28861820 10.1007/978-1-4939-7262-3_8

[59] Toyooka, K. & Kang, B. H. Reconstructing plant cells in 3D by serial section electron tomography. *Methods Mol. Biol.* **1080**, 159–170 (2014). 24132427 10.1007/978-1-62703-643-6_13

[60] Huang, L. et al. Endosidin20 targets the cellulose synthase catalytic domain to inhibit cellulose biosynthesis. *Plant Cell* **32**, 2141–2157 (2020). 32327535 10.1105/tpc.20.00202 PMC7346566

[61] Camacho, C. et al. BLAST+: architecture and applications. *BMC Bioinform.* **10**, 421 (2009). 10.1186/1471-2105-10-421 PMC2803857 20003500

[62] Edgar, R. C. MUSCLE: multiple sequence alignment with high accuracy and high throughput. *Nucleic Acids Res.* **32**, 1792–1797 (2004). 15034147 10.1093/nar/gkh340 PMC390337

[63] Castresana, J. Selection of conserved blocks from multiple alignments for their use in phylogenetic analysis. *Mol. Biol. Evol.* **17**, 540–552 (2000). 10742046 10.1093/oxfordjournals.molbev.a026334

[64] Minh, B. Q. et al. IQ-TREE 2: new models and efficient methods for phylogenetic inference in the genomic era. *Mol. Biol. Evol.* **37**, 1530–1534 (2020). 32011700 10.1093/molbev/msaa015 PMC7182206

[65] Letunic, I. & Bork, P. Interactive Tree of Life (iTOL) v4: recent updates and new developments. *Nucleic Acids Res.* **47**, W256–W259 (2019). 30931475 10.1093/nar/gkz239 PMC6602468

[66] Julian, J. ATG8ylation of vacuolar membrane protects plants against cell wall damage. *Zenodo*10.5281/zenodo.10993280 (2024). 10.1038/s41477-025-01907-z PMC11842276 39920307

