# Supplementary material for: ATG8ylation of vacuolar membrane protects plants against cell wall damage
Source: Nat Plants. 2025 Feb 7;11(2):321–39. doi: 10.1038/s41477-025-01907-z (PMC11842276; doi:10.1038/s41477-025-01907-z)
Supplement: Supplementary file 1 — Supplementary Methods. [file 41477_2025_1907_MOESM1_ESM.pdf]

nature plants

Supplementary information

https://doi.org/10.1038/s41477-025-01907-z

ATG8ylation of vacuolar membrane protects plants against cell wall damage

In the format provided by the authors and unedited

Supplementary Information – Material and Methods

Quantitative Real-Time PCR (qPCR)

Total RNA was extracted from 100 mg of tissue using the RNeasy Plant Mini Kit (Qiagen) following the manufacturer's instructions. RNA concentration and purity were assessed using a NanoDrop 2000 spectrophotometer (Thermo Fisher Scientific), with absorbance ratios A260/A280 and A260/A230 above 1.8 being accepted for further analysis.

One microgram of total RNA was treated with DNase I (Thermo Fisher Scientific) to eliminate genomic DNA contamination, and first-strand cDNA was synthesized using the RevertAid First Strand cDNA Synthesis Kit (Thermo Fisher Scientific) according to the manufacturer's protocol. Oligo(dT) primers were used to selectively synthesize cDNA from mRNA transcripts.

Primers were designed with melting temperatures (Tm) between 58–60°C and amplicon lengths of 80–150 bp. All primers were checked for secondary structures, including hairpins and primer dimers. Specificity was confirmed via BLAST analysis against the Arabidopsis genome. Primer efficiency was validated by constructing standard curves using a dilution series of cDNA.

FATG16Ex3	CACAAAGAAGGGAGGATTGTACACG
RATG16Ex4	CTTGTCATCTCCTCCAACTGAGACC
FATG16Ex5	CCGACATGTTGTCAGTGCAGC
RATG16Ex5.2	CCCATCCATGTGGCCTGAGA

qPCR was performed using the PowerUp SYBR Green Master Mix (Applied Biosystems) on a QuantStudio 6 Flex Real-Time PCR system (Thermo Fisher Scientific). Reactions were carried out in 96-well plates in a final volume of 10 µL, containing 5 µL SYBR Green Master Mix, 0.5 µL of each primer (final concentration 500 nM), 2 µL of diluted cDNA (corresponding to 50 ng total RNA), and 2 µL of nuclease-free water. The cycling conditions were as follows: 50°C for 2 minutes, 95°C for 10 minutes, followed by 40 cycles of 95°C for 15 seconds, 60°C for 1 minute. A melt curve analysis was included after the amplification cycle to verify the specificity of the PCR products. No-template controls were included in every run to confirm the absence of contamination.

The Ct (cycle threshold) values were normalized to the reference gene ACT2 (AT3G18780). The relative expression levels of target genes were calculated using the ΔΔCt method, where ΔCt represents the difference between the Ct values of the target gene and the reference gene, and ΔΔCt represents the difference between the experimental and control samples. Fold changes in gene expression were expressed as $2^{(-\Delta\Delta Ct)}$ and presented on a log2 scale.

Expression and purification of SidK in *E. coli*

For protein production in E. coli, amino acid residues Tyr9 to Asp278 from SidK WT and $SidK^{F62A}$ were amplified and subsequently cloned into pOPIN-GG vector pPGN-C (Bentham et al. 2021) with a N-terminal 6xHIS-3C and a C-terminal 3xFLAG via Golden Gate cloning [59].

Proteins were produced following established protocols for effector purification [60]. Briefly, plasmids were transformed into *E. coli* RosettaTM (DE3) and a single colony was grown overnight in LB media supplemented with appropriate antibiotics. Cell cultures were then grown in terrific broth media at 37°C for 5–7 hr and then at 16°C overnight. Cells were harvested by centrifugation and re-suspended in 50 mM Tris-HCl (pH 8), 500 mM NaCl, 5% (vol/vol) glycerol, and 20 mM imidazole supplemented with EDTA-free protease inhibitor tablets (Roche). Cells were sonicated and following centrifugation at 40,000xg for 30 min, the clarified lysate was applied to a HisTrapTM Ni2+-NTA column connected to an ÄKTA Pure chromatography system (Cytiva Life Sciences). Proteins were step-eluted with elution buffer (50 mM Tris-HCl (pH 8), 500 mM NaCl, 5% (vol/vol) glycerol, and 500 mM imidazole) and directly injected onto a Superdex 200 16/60 gel filtration column pre-equilibrated with 20 mM HEPES (pH 8), 150 mM NaCl and 5% (vol/vol) glycerol supplemented with 1mM TCEP. Elution fractions were collected and evaluated by SDS-PAGE. Relevant fractions were pooled together and concentrated as appropriate.

Affinity purification of biotinylated proteins and nanoLC-MS/MS Analysis

A. thaliana seeds were surface sterilized with ethanol, stratified for 2 days and grown in ½ MS (Duchefa)/0.5% MES/1% sucrose for 7 days under LEDs with 50 µmol m-2 s-1 and a 16 h light/8 h dark photoperiod. 7-days old seedlings were incubated with 50 µM biotin for 4 hours contextually with ES20-1 or Torin1 treatment, whereas Monensin-treated seedlings were incubated with 100 µM biotin for 2 hours. After the treatment, the seedlings were quickly rinsed in ice cold water, dried and frozen in liquid nitrogen. For the affinity purification of biotinylated proteins, around 1 gram of plant tissue was used for each sample and the protocol was performed as described by Mair et al. (2019) [66].

For MS Analysis, the nano HPLC system (UltiMate 3000 RSLC nano system) was coupled to an Orbitrap Exploris 480 mass spectrometer equipped with a Nanospray Flex ion source for the Exploris 480 (all parts Thermo Fisher Scientific). Peptides were loaded onto a trap column (PepMap Acclaim C18, 5 mm × 300 µm ID, 5 µm particles, 100 Å pore size, Thermo Fisher Scientific) at a flow rate of 25 µl/min using 0.1% TFA as mobile phase. After loading, the trap column was switched

in line with the analytical column (PepMap Acclaim C18, 500 mm × 75 μm ID, 2 μm, 100 Å, Thermo Fisher Scientific). Peptides were eluted using a flow rate of 230 nl/min, starting with the mobile phases 98% A (0.1% formic acid in water) and 2% B (80% acetonitrile, 0.1% formic acid) and linearly increasing to 35% B over the next 120 min. This was followed by a steep gradient to 95%B in 1 min, stayed there for 6 min and ramped down in 2 min to the starting conditions of 98% A and 2% B for equilibration at 30°C. The Orbitrap Exploris 480 mass spectrometer was operated in data-dependent mode 'Cycle Time', performing a full scan (m/z range 350-1200, resolution 60,000, normalized AGC target 300%, compensation voltages CV of -45V, -60V and -75V), followed by MS/MS scans of the most abundant ions for a cycle time of 0.9 seconds per CV. MS/MS spectra were acquired using an isolation width of 1.2 m/z, normalized AGC target 200%, HCD collision energy of 30, orbitrap resolution of 30,000, maximum injection time of 100 ms and minimum intensity set to 25,000. Precursor ions selected for fragmentation (include charge state 2-6) were excluded for 45 s. The monoisotopic precursor selection (MIPS) mode was set to Peptide and the exclude isotopes feature was enabled.

MS Data processing

For peptide identification, the RAW-files were loaded into Proteome Discoverer (version 2.5.0.400, Thermo Scientific). All MS/MS spectra were searched using MSAmanda v2.0.0.19924 [67]. The peptide mass tolerance was set to ±10 ppm and fragment mass tolerance to ±10 ppm, the maximum number of missed cleavages was set to 2, using tryptic enzymatic specificity without proline restriction. Peptide and protein identification was performed in two steps. For an initial search the RAW-files were searched against the Arabidopsis database called TAIR10 (32,785 sequences; 14,482,855 residues), supplemented with common contaminants and sequences of tagged proteins of interest using beta-methylthiolation on cysteine as a fixed modification. The result was filtered to 1 % FDR on protein level using the Percolator algorithm [68] integrated in Proteome Discoverer. A sub-database of proteins identified in this search was generated for further processing. For the second search, the RAW-files were searched against the created sub-database using the same settings as above and considering the following additional variable modifications: oxidation on methionine, deamidation on asparagine and glutamine, phosphorylation on serine, threonine and tyrosine, biotinylation on lysine, ubiquitinylation residue on lysine, glutamine to pyro-glutamate conversion at peptide N-terminal glutamine and acetylation on protein N-terminus. The localization of the post-translational modification sites within the peptides was performed with the tool ptmRS, based

on the tool phosphoRS [69]. Identifications were filtered again to 1 % FDR on protein and PSM level, additionally an Amanda score cut-off of at least 150 was applied. Proteins were filtered to be identified by a minimum of 2 PSMs in at least 1 sample. Protein areas have been computed in IMP-apQuant [70] by summing up unique and razor peptides. Resulting protein areas were normalised using iBAQ [71] and sum normalisation was applied for normalisation between samples.

For quality control, proteins with PSMs >= 5 were filtered for each Torin-treated sample. Known ATG8 interactors specifically enriched in TID-ATG8A Torin sample are shown in Fig. 4d. Proteins with log2FC >=1 in TID-ATG8 samples, respectively to the correspondent treatment in TID control, were filtered to identify ATG8 interactors upon ES20-1 and Monensin treatment, shown in Fig. 4b-d. Venn diagrams, volcano plots and dot plots were produced with eulerr R package version 6.1.1 (https://CRAN.R-project.org/package=eulerr).

Bibliography

[59]Engler, C., Kandzia, R. & Marillonnet, S. A one pot, one step, precision cloning method with high throughput capability. *PLoS ONE* **3**, e3647 (2008).

[60]De la Concepcion, J. C. et al. A blast fungus zinc-finger fold effector binds to a hydrophobic pocket in host Exo70 proteins to modulate immune recognition in rice. *Proc. Natl Acad. Sci. USA 119*, e2210559119 (2022).

[61]Bentham, R. et al.Using DNA sequencing data to quantify T cell fraction and therapy response. *Nature* **597**(7877):555-560 (2021).

[66]Mair, A., Xu, S.-L., Branon, T. C., Ting, A. Y. & Bergmann, D. C. Proximity labeling of protein complexes and cell-type-specific organellar proteomes in Arabidopsis enabled by TurboID. *eLife* **8**, e47864 (2019).

[67]Dorfer, V. et al. MS Amanda, a universal identification algorithm optimized for high accuracy tandem mass spectra. *J. Proteome Res.* **13**, 3679–3684 (2014).

[68]Käll, L., Canterbury, J. D., Weston, J., Noble, W. S. & MacCoss, M. J. Semi-supervised learning for peptide identification from shotgun proteomics datasets. *Nat. Methods* **4**, 923–925 (2007).

[69]Taus, T. et al. Universal and confident phosphorylation site localization using phosphoRS. *J. Proteome Res.* **10**, 5354–5362 (2011).

[70]Doblmann, J. et al. apQuant: accurate label-free quantification by quality filtering. *J. Proteome Res*. **18**, 535–541 (2019).

[71]Schwanhäusser, B. et al. Global quantification of mammalian gene expression control. *Nature* **473**, 337–342 (2011).
