# Supplementary material for: ATG8ylation of vacuolar membrane protects plants against cell wall damage
Source: Nat Plants. 2025 Feb 7;11(2):321–39. doi: 10.1038/s41477-025-01907-z (PMC11842276; doi:10.1038/s41477-025-01907-z)
Supplement: Supplementary file 2 — Reporting Summary [file 41477_2025_1907_MOESM2_ESM.pdf]

nature portfolio

Corresponding author(s): Yasin Dagdas
Jose Julian Valenzuela

Last updated by author(s): Nov 20, 2024

Reporting Summary

Nature Portfolio wishes to improve the reproducibility of the work that we publish. This form provides structure for consistency and transparency in reporting. For further information on Nature Portfolio policies, see our Editorial Policies and the Editorial Policy Checklist.

Statistics

For all statistical analyses, confirm that the following items are present in the figure legend, table legend, main text, or Methods section.

n/a	Confirmed	
☐	☒	The exact sample size (*n*) for each experimental group/condition, given as a discrete number and unit of measurement
☐	☒	A statement on whether measurements were taken from distinct samples or whether the same sample was measured repeatedly
☐	☒	The statistical test(s) used AND whether they are one- or two-sided<br>*Only common tests should be described solely by name; describe more complex techniques in the Methods section.*
☒	☐	A description of all covariates tested
☒	☐	A description of any assumptions or corrections, such as tests of normality and adjustment for multiple comparisons
☐	☒	A full description of the statistical parameters including central tendency (e.g. means) or other basic estimates (e.g. regression coefficient) AND variation (e.g. standard deviation) or associated estimates of uncertainty (e.g. confidence intervals)
☐	☒	For null hypothesis testing, the test statistic (e.g. *F*, *t*, *r*) with confidence intervals, effect sizes, degrees of freedom and *P* value noted<br>*Give P values as exact values whenever suitable.*
☒	☐	For Bayesian analysis, information on the choice of priors and Markov chain Monte Carlo settings
☐	☒	For hierarchical and complex designs, identification of the appropriate level for tests and full reporting of outcomes
☐	☒	Estimates of effect sizes (e.g. Cohen's *d*, Pearson's *r*), indicating how they were calculated

Our web collection on statistics for biologists contains articles on many of the points above.

Software and code

Policy information about availability of computer code

Data collection	Proteome Discoverer 2.5, ZEISS ZEN Blue 3.9, SymPhoTime 64 2.9, Serial EM 4.1
Data analysis	MSAmanda v2.0.0.19924, eulerr R package version 6.1.1, IMP-apQuant v2.5, CRISPR-P 2.0, Fiji/ImageJ v1.54f, IQ-Tree 2 1.6.11, Gblocks 2, BLAST+ 2.15.0, iToL 6.9, ptmRS, IMOD 10.1

For manuscripts utilizing custom algorithms or software that are central to the research but not yet described in published literature, software must be made available to editors and reviewers. We strongly encourage code deposition in a community repository (e.g. GitHub). See the Nature Portfolio guidelines for submitting code & software for further information.

Data

Policy information about availability of data

All manuscripts must include a data availability statement. This statement should provide the following information, where applicable:

- Accession codes, unique identifiers, or web links for publicly available datasets
- A description of any restrictions on data availability
- For clinical datasets or third party data, please ensure that the statement adheres to our policy

All the source data used to generate the main and supplementary figures are deposited to Zenodo (10.5281/zenodo.10993280). The Arabidopsis reference genome was obtained from TAIR10 (https://www.arabidopsis.org). Other reference genomes were obtained from Phytozome 13 (https://phytozome-next.jgi.doe.gov/) and Solanaceae Genomics Network (https://solgenomics.sgn.cornell.edu/).

Research involving human participants, their data, or biological material

Policy information about studies with human participants or human data. See also policy information about sex, gender (identity/presentation), and sexual orientation and race, ethnicity and racism.

Reporting on sex and gender	n/a
Reporting on race, ethnicity, or other socially relevant groupings	n/a
Population characteristics	n/a
Recruitment	n/a
Ethics oversight	n/a

Note that full information on the approval of the study protocol must also be provided in the manuscript.

Field-specific reporting

Please select the one below that is the best fit for your research. If you are not sure, read the appropriate sections before making your selection.

☒ Life sciences ☐ Behavioural & social sciences ☐ Ecological, evolutionary & environmental sciences

For a reference copy of the document with all sections, see nature.com/documents/nr-reporting-summary-flat.pdf

Life sciences study design

All studies must disclose on these points even when the disclosure is negative.

Sample size	Sample sizes were selected based on guidance from prior literature and experimental logistics. Similar studies examining the vacuole or other organelles in Arabidopsis thaliana (e.g., Dunser et al., 2019, EMBO J; Cui et al., 2019, Nature Plants) used comparable sample sizes to ensure robust data acquisition while accommodating practical constraints. Additionally, the consistency of results within and between replicates was closely monitored, and the variability observed was minimal. This confirmed that our chosen sample size was sufficient to draw reliable conclusions for the scope of this study.
Data exclusions	No data were excluded.
Replication	All experiments (Western blots, coIP and mass spec analysis, carbon starvation assays, mechanoprobe sensors measurements, qPCRs and the TurboID) in this study were measured at least 3 times with comparable results and no unsuccessful replication.<br>For all confocal microscopy imaging at least 10 biological replicas were performed. All attempt at replication were successful.<br>For EM microscopy at least 3 biological replicates were performed. All attempt at replication were successful.
Randomization	No experimental group was used in this study. In the carbon starvation assay, to avoid possible positional and developmental effectsn, each genotype to be tested was grown in a different position of the multi well plate for each replica.
Blinding	Blinding was not performed in this study because the experimental design inherently minimized the risk of bias. All images and raw data from all samples were acquired and stored systematically, enabling transparent re-analysis at any time. Additionally, the nature of the experiments —such as the use of predefined, quantifiable metrics for image analysis (e.g., puncta counting, vacuolar morphology index)—limited the potential for subjective interpretation. The consistency of results within and between replicates further supports the reliability of our conclusions. If necessary, stored data could be scored independently by additional researchers to confirm findings.

Reporting for specific materials, systems and methods

We require information from authors about some types of materials, experimental systems and methods used in many studies. Here, indicate whether each material, system or method listed is relevant to your study. If you are not sure if a list item applies to your research, read the appropriate section before selecting a response.

Materials & experimental systems

n/a	Involved in the study
☐	☒ Antibodies
☒	☐ Eukaryotic cell lines
☒	☐ Palaeontology and archaeology
☒	☐ Animals and other organisms
☒	☐ Clinical data
☒	☐ Dual use research of concern
☐	☒ Plants

Methods

n/a	Involved in the study
☒	☐ ChIP-seq
☒	☐ Flow cytometry
☒	☐ MRI-based neuroimaging

Antibodies

Antibodies used

(1) GFP was detected using the anti-GFP antibody (Mouse monoclonal, IgG1κ (clones 7.1 and 13.1), 11814460001; Roche) diluted 1:5000 (v/v).
(2) NBR1 was detected using the anti-NBR1 antibody (Rabbit polyclonal, AS14 2805; Agrisera) diluted 1:5000 (v/v).
(3) The subunit A of the V-ATPase was detected using the anti-V-ATPase-A antibody (Rabbit polyclonal, AS09 467; Agrisera) diluted 1:5000 (v/v).
(4) FLAG was detected using anti-FLAG M2 antibody (Mouse monoclonal, M2 clone, F3165, Sigma) diluted to 10 μg/mL.
(5) mCherry was detected using anti-RFP (Mouse monoclonal, IgG2c 6G6 clone, AB_2631395, Chromotek) diluted 1:2000 (v/v).
Mouse monoclonal antibodies were detected with goat anti-mouse IgG HRP-linked antibody (61-6520; Invitrogen) diluted 1:5000 (v/v).
Rabbit polyclonal antibody was detected with a goat anti-rabbit IgG HRP-linked antibody (65-6120; Invitrogen) diluted 1:5000.

Validation

All the antibodies used in this study were commercial antibodies to standard epitope tags, validated by manufacturers. Reference papers provided by manufacturers:
(1) Chi C Wong et al. Blood, 118(16), 4305-4312 (2011-08-02)
(2) Mosesso et al. Nat Commun. 2024 Jun 19;15(1):5188. doi: 10.1038/s41467-024-49485-6.
(3) Vera-Estrella et al. Planta. 2017 Apr 28. doi: 10.1007/s00425-017-2700-1.
(4) Brizzard, B.L., et al. BioTechniques, 16, 730-735 (1994)
(5) Jha el al. Nature. 2017 Jun 29;546(7660):651-655. doi: 10.1038/nature22814

Plants

Seed stocks

No plant specimen was collected from the field. All seed stocks are described in Supplementary Table S3.
pUbi::mCherry-ATG8A At4g21980 Stephani et al. (2020) eLife.
pUbi::GFP-ATG8A At4g21980 Stephani et al. (2020) eLife.
pUBQ::GFP-ATG8B At4g04620 Stephani et al. (2020) eLife.
pUBQ::GFP-ATG8C At1g62040 Stephani et al. (2020) eLife.
pUBQ::GFP-ATG8D At2g05630 Stephani et al. (2020) eLife.
pUBQ::GFP-ATG8E At2g45170 Stephani et al. (2020) eLife.
pUBQ::GFP-ATG8F At4g21980 Stephani et al. (2020) eLife.
pUBQ::GFP-ATG8G At3g60640 Stephani et al. (2020) eLife.
pUBQ::GFP-ATG8H At3g06420 Stephani et al. (2020) eLife.
pUBQ::GFP-ATG8I At3g15580 Stephani et al. (2020) eLife.
pUBQ::mCherry-CFS1 At3g43230 Zhao et al. (2022), J Cell Bio.
GFP-ATG8A x mCherry-CFS1 At4g21980 (ATG8A) Zhao et al. (2022), J Cell Bio.
At3g43230 (CFS1) pUBQ::VAMP711-YFP At4g32150 Geldner et al. (2009) Plant J.
At4g32150 (VAM711) pNBR1::NBR1-GFP At4g24690 Hafren et al. (2017) PNAS.
mCherry-ATG8F NBR1-GFP At4g21980 (NBR1) At2g45170 (ATG8F) Hafren et al. (2017) PNAS.
g-TIP-GFP At2g36830 Brownfield et al. (2015) Nature
atg5-1 At5g17290 SAIL_129B07L, Thompson et al. (2005) Plant Physiol.
atg11 At4g30790 Li et al. (2014) Plant Cell.
fer-4 and frx3/4/5 Dunser et al. (2019) EMBO J.
GFP-FREE1 At1g20110 Gao et al., 2014, Current Biology.
GFP-ALIX AT1g15130 Garcia-Leon (2019) Plant Cell
pVPS23A::VPS23A-RFP At3g12400 Nagel et al., 2017, PNAS
pEF::GFP-MpATG8A Mp1g21590 Noruzuki et al., 2019, Frontiers i.
pEF::GFP-MpATG8B Mp5g05930 Noruzuki et al., 2019, Frontiers i.
GFP-ATG8F At2g45170 Zou et al. (2024) Bioarxiv
GFP-ATG8F in atg4 At4g21980(ATG8A) At2g45170 (ATG8F) Zou et al. (2024) Bioarxiv
GFP-ATG8I AT3G15580 Zou et al. (2024) Bioarxiv
GFP-ATG8I in atg4 At4g21980(ATG8A) AT3G15580 (ATG8I) Zou et al. (2024) Bioarxiv

Novel plant genotypes

All Arabidopsis thaliana lines used in this study originate from the Columbia (Col0) ecotype background and are listed in Supplementary Table S3. All transgenic lines were generated by floral dipping and plasmid constructs were cloned with the GreenGate cloning method. The coding sequence of genes of interest were ordered from Twist Biosciences. For DEX-induced expression of SidK in Arabidopsis roots, full length sequence of SidK or SidKF62A were synthesized (Twist) and subsequently cloned by GreenGate cloning with a C-terminal mCherry tag under control of 3xOPp into Module N. GR-LhG4 expression is driven by UBI10 promoter and cloned in Module M. Module M and N were combined in the destination vector pGGZ003 and transformed in Arabidopsis following Agrobacterium-mediated standard protocols. APEX2-ATG8A was cloned by GreenGate cloning into pGGSun with a UBI10 promoter. Homozygous plants with similar expression levels of APEX2 were selected. The knock-out mutant atg16 and the truncation mutant ACASM were created by CRISPR/Cas9-mediated mutation. The plasmid pCBCDT1T2 was used as scaffold template and pHEE401E as destination vector. The guides, designed via CRISPR-P 2.0 (http://crispr.hzau.edu.cn/cgi-bin/CRISPR2/CRISPR) are GATCGGGAAACCATTGGCAT and GGTACAGGAGGAGAAAGCTA for atg16 and GATCGGGAAACCATTGGCAT and GATCGGTTCCATCTTCATTG for ACASM. Cas9 was crossed out. ATG16 was cloned by GreenGate cloning into pGGZ003 with a UBI10 promoter. ATG16 complementation constructs were cloned by PCR from the previous vector. Each PCR (ATG161-195, ATG161-253, ATG161-295, ATG161-379) became a module which was cloned by GreenGate cloning into pGGSun with a UBI10 promoter. ATG16 and the four other delta C deletions were transformed into the atg16 mutant background and homozygous plants were selected. Male Marchantia polymorpha Takaragaike-1 (Tak-1) plants were maintained asexually and cultured through gemma on half-strength Gamborg's B5 medium supplemented with 0.5 g/l MES, 1% sucrose and 1% agar under 50 μM/m2/s continuous white light at 21 °C.

Authentication

All novel lines were tested by PCR and by western or by directly seeing the signal in the confocal microscope. All previously described lines were test to behave as they did in their original paper.
