# Supplementary figures and images for: ATG8ylation of vacuolar membrane protects plants against cell wall damage
Source: Nat Plants. 2025 Feb 7;11(2):321–39. doi: 10.1038/s41477-025-01907-z (PMC11842276; doi:10.1038/s41477-025-01907-z)

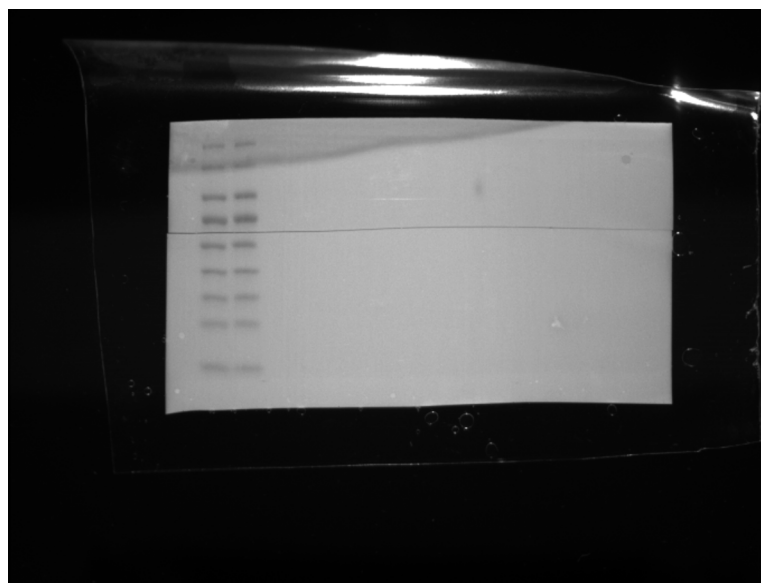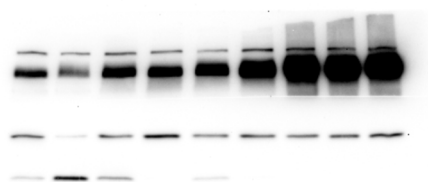

Supplement: Supplementary file 6 — Unprocessed western blots. [file 41477_2025_1907_MOESM6_ESM.pdf]

D

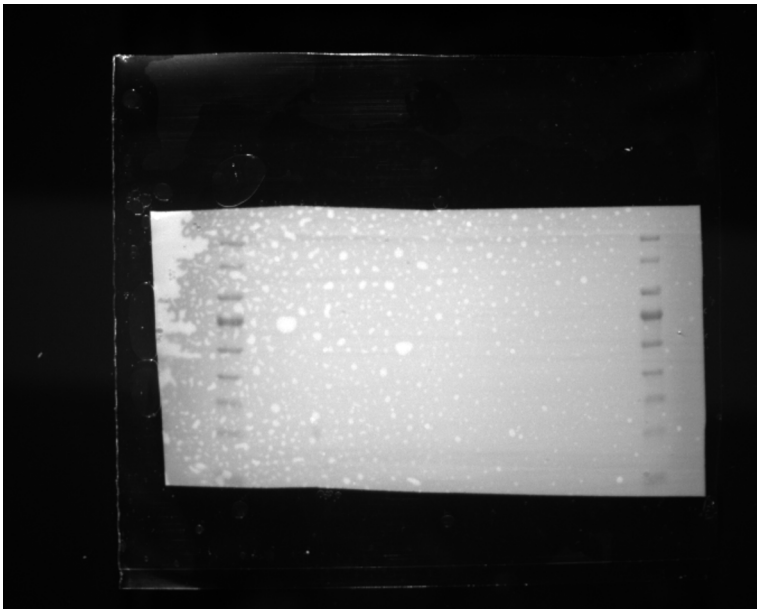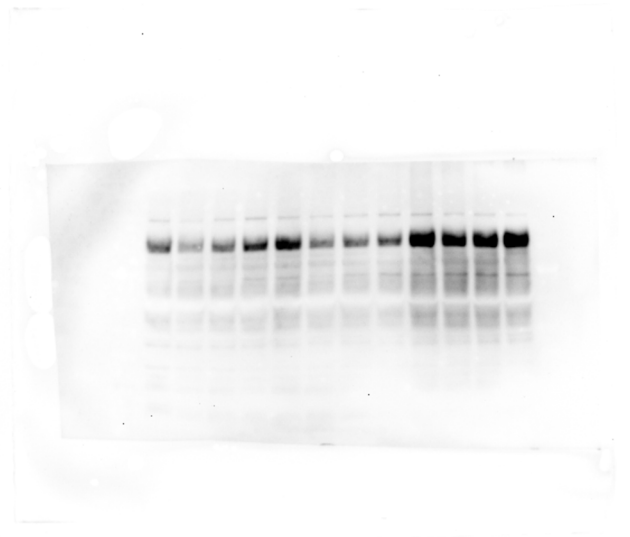

E

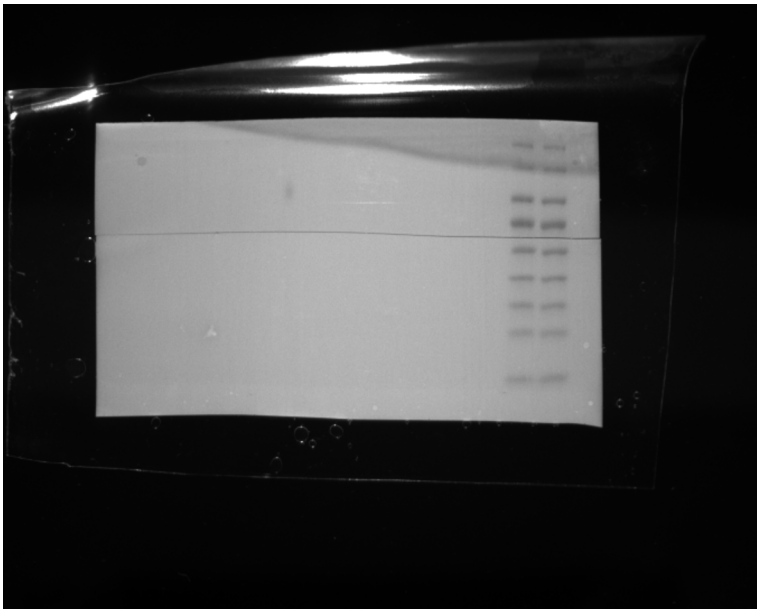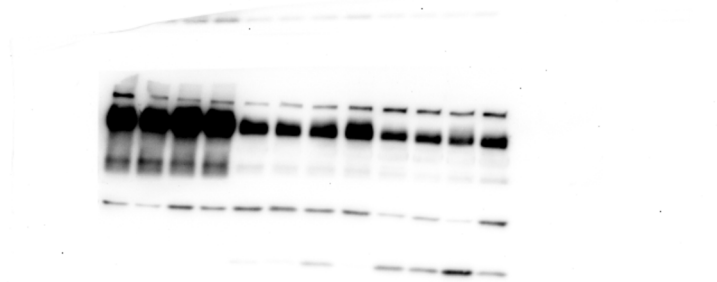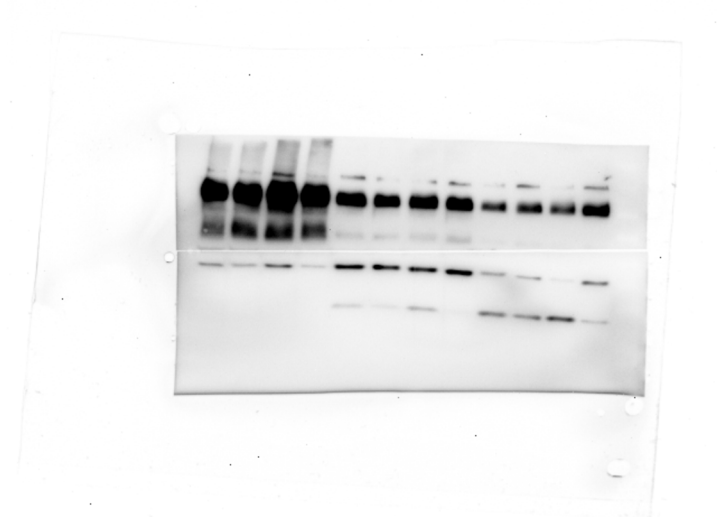

Supplement: Supplementary file 7 — Unprocessed western blots. [file 41477_2025_1907_MOESM7_ESM.pdf]

A

input A

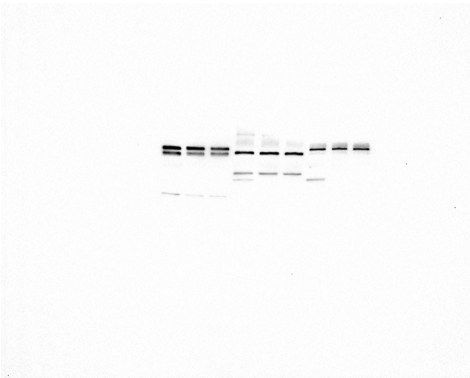

input FLAG

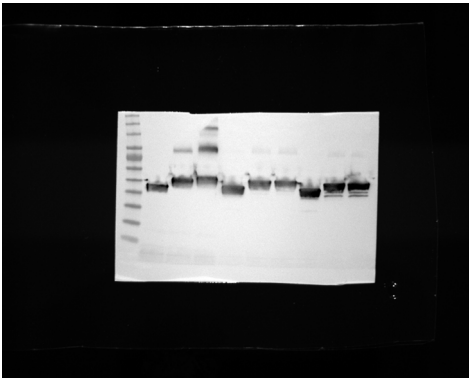

PS

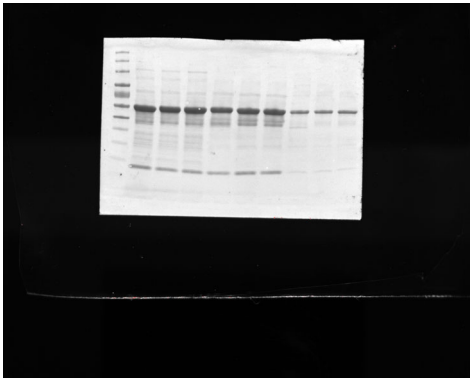

IP A

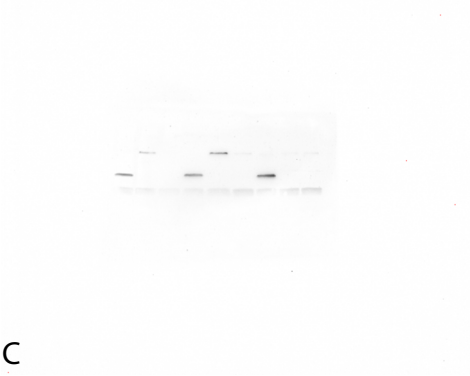

IP FLAG

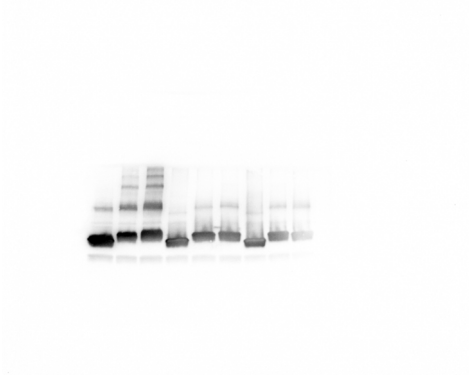

C

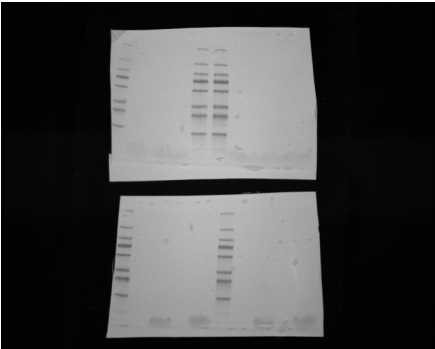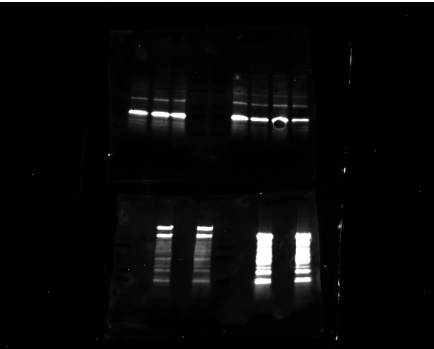

Supplement: Supplementary file 8 — Unprocessed western blots. [file 41477_2025_1907_MOESM8_ESM.pdf]

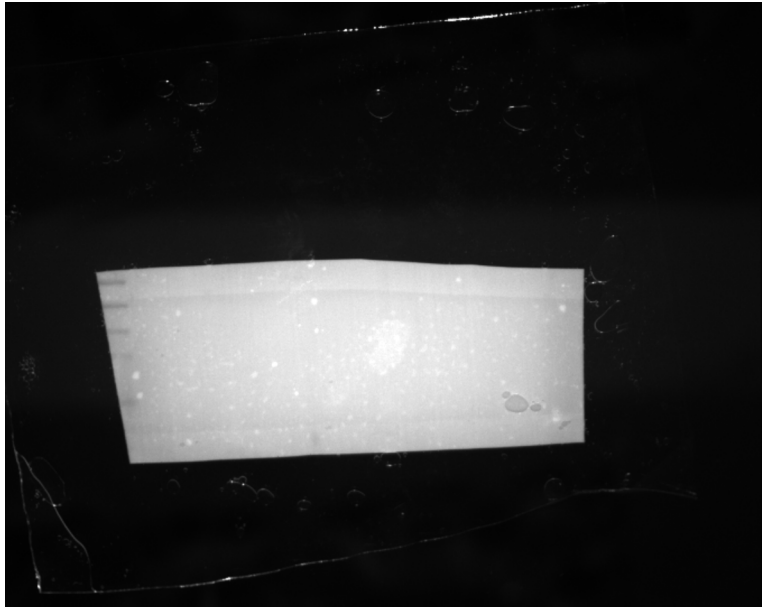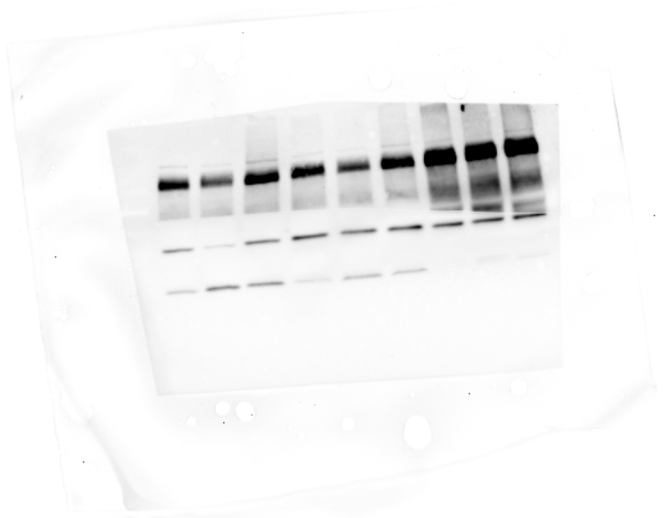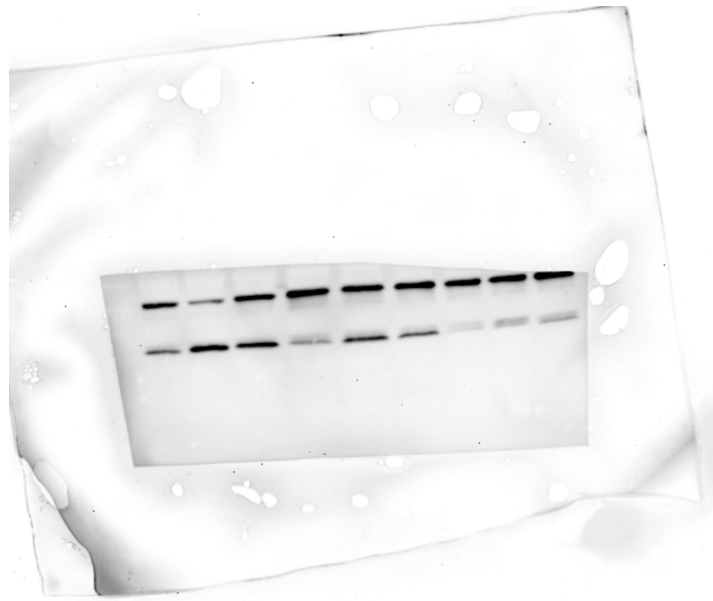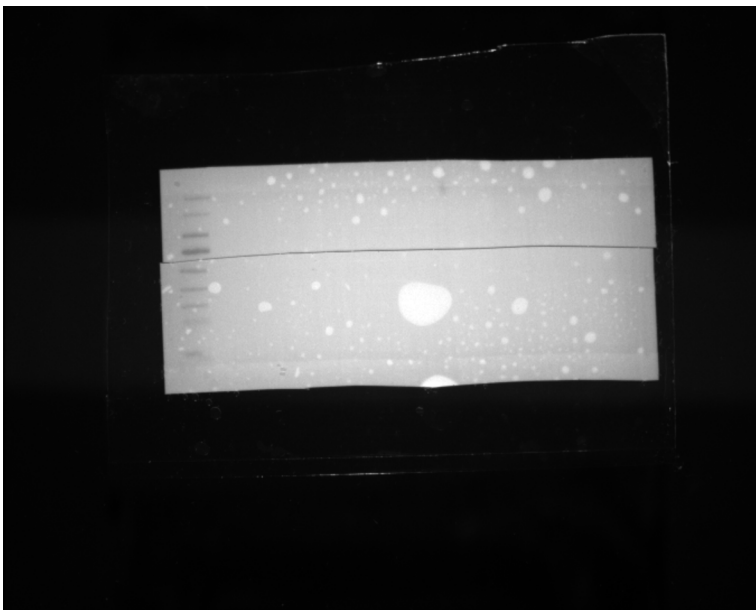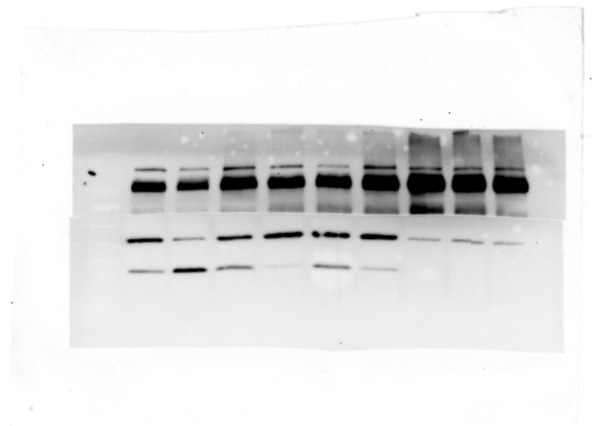

Supplement: Supplementary file 9 — Unprocessed western blots. [file 41477_2025_1907_MOESM9_ESM.pdf]
